# Interactome Analysis Identifies MSMEI_3879 as a Substrate of Mycolicibacterium smegmatis ClpC1

**DOI:** 10.1128/spectrum.04548-22

**Published:** 2023-06-21

**Authors:** Emmanuel C. Ogbonna, Henry R. Anderson, Patrick C. Beardslee, Priyanka Bheemreddy, Karl R. Schmitz

**Affiliations:** a Department of Biological Sciences, University of Delaware, Newark, Delaware, USA; b Department of Chemistry and Biochemistry, University of Delaware, Newark, Delaware, USA; The Pennsylvania State University

**Keywords:** Clp protease, *Mycobacterium*, interactome, proteolysis, proteomics

## Abstract

The prevalence of drug-resistant Mycobacterium tuberculosis infections has prompted extensive efforts to exploit new drug targets in this globally important pathogen. ClpC1, the unfoldase component of the essential ClpC1P1P2 protease, has emerged as one particularly promising antibacterial target. However, efforts to identify and characterize compounds that impinge on ClpC1 activity are constrained by our limited knowledge of Clp protease function and regulation. To expand our understanding of ClpC1 physiology, we employed a coimmunoprecipitation and mass spectrometry workflow to identify proteins that interact with ClpC1 in Mycolicibacterium smegmatis, a surrogate for M. tuberculosis. We identify a diverse panel of interaction partners, many of which coimmunoprecipitate with both the regulatory N-terminal domain and the ATPase core of ClpC1. Notably, our interactome analysis establishes MSMEI_3879, a truncated gene product unique to M. smegmatis, as a novel proteolytic substrate. Degradation of MSMEI_3879 by ClpC1P1P2 *in vitro* requires exposure of its N-terminal sequence, reinforcing the idea that ClpC1 selectively recognizes disordered motifs on substrates. Fluorescent substrates incorporating MSMEI_3879 may be useful in screening for novel ClpC1-targeting antibiotics to help address the challenge of M. tuberculosis drug resistance.

**IMPORTANCE** Drug-resistant tuberculosis infections are a major challenge to global public health. Much effort has been invested in identifying new drug targets in the causative pathogen, Mycobacterium tuberculosis. One such target is the ClpC1 unfoldase. Compounds have been identified that kill M. tuberculosis by disrupting ClpC1 activity, yet the physiological function of ClpC1 in cells has remained poorly defined. Here, we identify interaction partners of ClpC1 in a model mycobacterium. By building a broader understanding of the role of this prospective drug target, we can more effectively develop compounds that inhibit its essential cellular activities.

## INTRODUCTION

Tuberculosis is responsible for greater global mortality than any other bacterial pathogen, causing an estimated 1.5 million deaths in 2020 alone (1). While rates of infection and mortality have declined over the past decade, the prevalence of multidrug-resistant tuberculosis infections remains a persistent challenge to global public health. There is consequently an urgent need to develop new drugs and exploit new drug targets in the causative pathogen, Mycobacterium tuberculosis. The essential mycobacterial Clp proteases have emerged as one promising class of targets (2–7).

Clp proteases are well-studied proteolytic machines that unravel and hydrolyze native protein substrates (8, 9). These large oligomeric complexes consist of a hexameric Clp unfoldase that recognizes substrates, unfolds them using energy from ATP hydrolysis, and spools them into an associated peptidase barrel for degradation (10). The mycobacterial Clp peptidase is composed of two paralogous heptamers, ClpP1 and ClpP2, that assemble into a catalytically active hetero-oligomeric ClpP1P2 tetradecamer (11–16). Two alternative unfoldases, ClpC1 and ClpX, dock on the ClpP2 face of the peptidase to form the functional Clp protease (13, 14, 17, 18). Substrates are recognized by directly interacting with the unfoldase (19–21), with the aid of proteolytic adaptors (22, 23), or via posttranslational phosphorylation (24–26).

All components of M. tuberculosis Clp proteases are strictly essential for viability (19, 27–31), making these enzymes attractive targets for novel drug development. Multiple classes of antimicrobials inhibit or dysregulate Clp protease activity by targeting the peptidase (2, 5, 13, 14, 29, 32–34). However, several compound classes have been shown to cross-target human mitochondrial CLPP (35–37), which complicates efforts to develop ClpP-targeting pharmacophores into viable antibacterial therapeutics. By contrast, ClpC1 has no direct homologs in animals, and thus provides an avenue for inhibiting Clp protease activity in the pathogen with lower risk of off-target effects in humans. Cyclic peptides have been identified that kill M. tuberculosis by targeting ClpC1, including cyclomarin A, metamarin, lassomycin, ecumicin (ECU) and rufomycin (RUF), all of which bind to the N-terminal domain (NTD) (3, 4, 38–43).

Bacterial Clp proteases participate in various physiological processes, including protein quality control, response to stress, and regulation of virulence (19, 44–53). Although Clp proteases are known to be essential in mycobacteria, the breadth of their physiological roles is poorly understood. One path to elucidating the functions of Clp proteases is to identify their proteolytic substrates and interaction partners. Prior studies have used targeted capture approaches to identify Clp protease substrates in several bacteria, including Escherichia coli, Bacillus subtilis, Staphylococcus aureus, and Caulobacter crescentus (48, 52, 54–56).

In this study, we use coimmunoprecipitation and mass spectrometry to identify cellular proteins that interact with the ClpC1 unfoldase in Mycolicibacterium smegmatis, a nonpathogenic relative of M. tuberculosis. Importantly, we identify and characterize a novel substrate of the ClpC1P1P2 protease in M. smegmatis.

## RESULTS

### Identification of interaction partners of full-length ClpC1.

**FIG 1:**
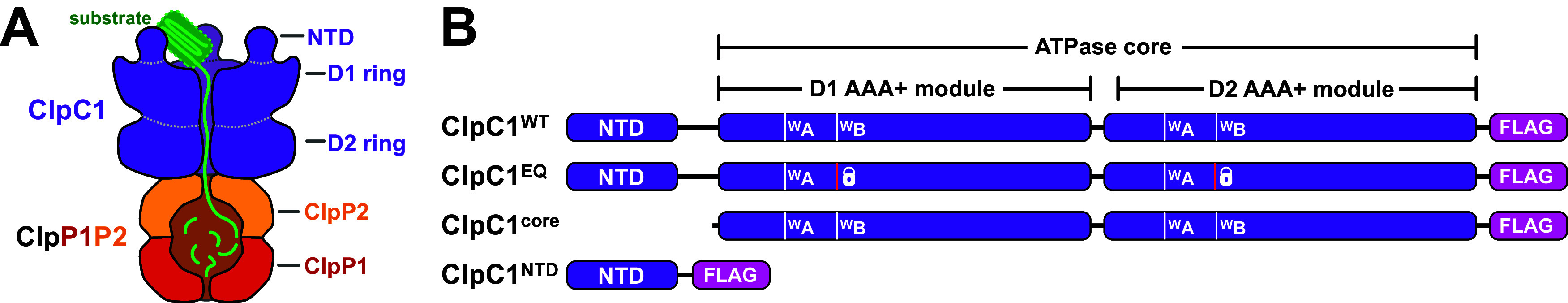
A ClpC1 Walker B double mutant substrate trap was generated in Mycolicibacterium smegmatis (strain ATCC 700084/MC^2^155). (A) General architecture of the mycobacterial Clp protease. The ATP-dependent unfoldase ClpC1 is responsible for recognizing (via the NTD) and unfolding protein substrates in an ATP-dependent manner (via AAA+ domains D1 and D2), and translocating them into the peptidase barrel composed of catalytic heptamers ClpP1 and ClpP2, wherein degradation occurs. ClpC1^EQ^ associates stably with protein substrates but is unable to unfold or translocate them. (B) Linear sequence models showing the ClpC1 constructs used in this study to identify cellular proteins interacting with specific ClpC1 components such as the NTD and AAA+ core.

We sought to design a set of ClpC1 constructs that maximized our likelihood of identifying diverse interaction partners. Some interaction partners likely bind to wild-type ClpC1 (ClpC1^WT^) irrespective of its nucleotide-bound state: for example, to the flexible NTD. However, substrate binding to wild-type AAA+ unfoldases is often transient, due to unfolding/degradation, and ATP dependent (8, 9, 57–59). To increase our ability to capture ATP-dependent interactions and prevent degradation of bona fide substrates, we mutated conserved Glu residues within the D1 and D2 Walker B motifs to Gln (E288Q and E626Q, respectively), yielding ClpC1^EQ^ (Fig. 1A and B) (60). Analogous mutations in related AAA+ enzymes stabilize ATP binding but impair nucleotide hydrolysis and substrate unfolding (56, 61, 62). To facilitate coimmunoprecipitation with interaction partners, all constructs incorporated a C-terminal 3×FLAG tag (DYKDHDG-DYKDHDI-DYKDDDDK). We confirmed that addition of the C-terminal tag did not impair the ATPase activity of ClpC1 or protease activity of ClpC1P1P2 *in vitro* (see Fig. S1A and B in the supplemental material). We additionally confirmed that that FLAG-tagged ClpC1 can substitute for endogenous ClpC1 in M. smegmatis, albeit with a growth defect (Fig. S1C to H), indicating that this construct retains at least partial ability to support proteolysis in cooperation with ClpP1P2.

**FIG 2:**
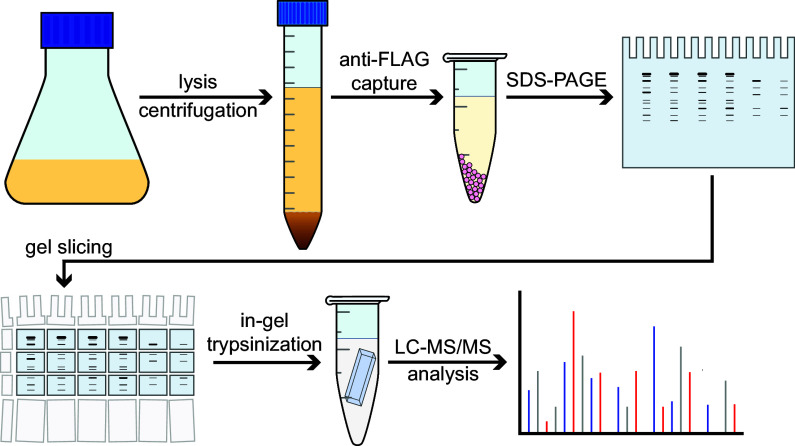
Workflow for identification of ClpC1 interaction partners. Mycolicibacterium smegmatis (ATCC 700084/MC^2^155) cells were grown up to and *A*_600_ of ≈0.6 to 1.0, and ClpC1 expression was induced following addition of 28 mM ε-caprolactam. Identification of the interactome was based on gel electrophoresis, trypsin digestion, and LC-MS/MS run using higher-energy collisional dissociation (HCD) fragmentation. All ClpC1 test construct and control experiments were subjected to the same workflow and were carried out in biological replicates.

**FIG 3:**
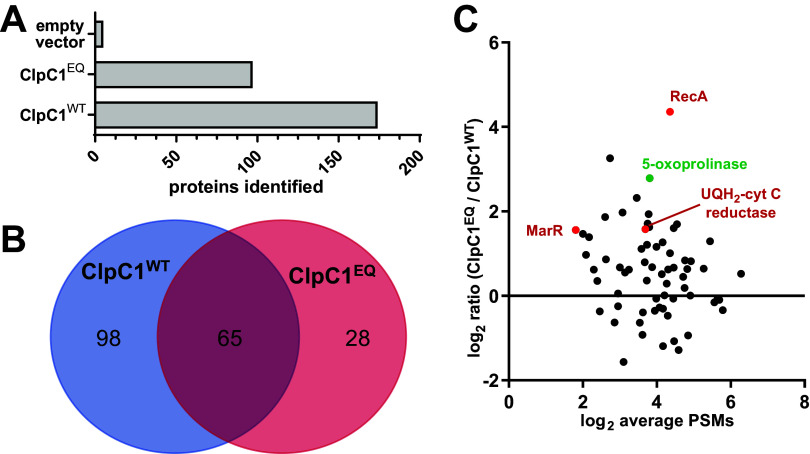
Comparative analysis of the ClpC1^EQ^ and ClpC1^WT^ interactome. (A) Bar graph showing the number of proteins identified in the ClpC1^EQ^ and ClpC1^WT^ data sets; (B) Venn diagram showing the number of Mycolicibacterium smegmatis cellular proteins that interacted with ClpC1^EQ^ and ClpC1^WT^ constructs, respectively. The numbers in panels A and B exclude ClpC1 itself. (C) Volcano plot showing enrichment of proteins captured in the M. smegmatis ClpC1 mutant trap versus wild type by liquid chromatography-tandem mass spectrometry (LC-MS/MS). The *y* axis shows the log_2_ ratio of normalized scores in the ClpC1^EQ^ data set to those in the ClpC1^WT^ data set, while the *x* axis shows log_2_ average number of PSMs for the proteins. Red circles represent proteins enriched in ClpC1^EQ^ with a *P* value of <0.05 by Student’s *t* test.

ClpC1 constructs, or an empty vector control, were expressed in M. smegmatis MC^2^155. ClpC1 and interaction partners were coimmunoprecipitated from lysates in the presence of 10 mM ATP using anti-FLAG beads and separated by SDS-PAGE (Fig. 2). Samples were prepared by in-gel trypsin digestion and analyzed by liquid chromatography-tandem mass spectrometry (LC-MS/MS) using higher-energy collisional dissociation (HCD) fragmentation. Proteomic analysis was performed in the Proteome Discoverer software suite against the M. smegmatis MC^2^155 proteome using Sequest HT. Proteins observed in at least two of three biological replicates were considered for further analysis. Five proteins were observed in the empty vector control (Fig. 3A), presumably due to nonspecific interactions with beads. Occurrences of these proteins were ignored in all other samples. In total, 163 and 93 proteins were identified in ClpC1^WT^ and ClpC1^EQ^ data sets, respectively (Fig. 3A; see Tables S1 and S2 in the supplemental material).

We expected substrates to bind more stably to ClpC1^EQ^ than to ClpC1^WT^ because the wild-type enzyme can presumably unfold substrates in the pulldown conditions. Within the ClpC1^EQ^ data set, 28 proteins matched these criteria and were absent from the ClpC1^WT^ data (Fig. 3B and Table 1). These include cell wall synthesis protein Wag31, succinate dehydrogenase iron-sulfur protein SdhB, aconitate hydratase A (AcnA), and ATP synthase epsilon chain AtpC. There were 65 proteins observed in both the ClpC1^WT^ and ClpC1^EQ^ data sets (Fig. 3A and B). A semiquantitative comparison indicates that 20 of these were enriched in ClpC1^EQ^ over ClpC1^WT^ by at least a ratio of 2:1 in average normalized score (Fig. 3C; Table S2). However, only three proteins were enriched significantly (with a *P* value of <0.05): the DNA repair and stress response protein RecA, electron transport chain component ubiquinol-cytochrome *c* reductase cytochrome *b* subunit (MSMEG_4263), and a MarR family protein transcriptional regulator (MSMEG_2538) (Fig. 3B). The co-occurrence of these in ClpC1^WT^ and ClpC1^EQ^ data sets suggests that these interactions occur stably regardless of ATPase and unfolding activity, as would be expected for adaptors, regulators, or other non-substrate interactors.

**TABLE 1:** Proteins that coimmunoprecipitate with ClpC1^EQ^ but not ClpC1^WT^

UniProt accession no.	Gene name(s)	Protein description	Normalized avg score^*a*^	Avg no. of PSMs
A0R461	MSMEG_5715, MSMEI_5564	Bac_luciferase domain-containing protein	0.390	10.667
A0QWT6	MSMEG_3058, MSMEI_2982	Lipoprotein	0.144	3.000
A0QSP8	*rplM*, MSMEG_1556, MSMEI_1519	50S ribosomal protein L13	0.085	6.667
A0QSD2	*rplD*, MSMEG_1437, MSMEI_1401	50S ribosomal protein L4	0.048	5.000
A0QRD2	MSMEG_1073, MSMEI_1041	Oxidoreductase, short-chain dehydrogenase/reductase family protein	0.091	9.333
A0R1Z9	*atpC*, MSMEG_4935, MSMEI_4808	ATP synthase epsilon chain (ATP synthase F1 sector epsilon subunit)	0.060	3.000
A0QVU2	MSMEG_2695, MSMEI_2629	35-kDa protein	0.083	2.500
A0QWK5	*ppiB*, MSMEG_2974, MSMEI_2900	Peptidyl-prolyl *cis*-*trans* isomerase (PPIase)	0.085	2.500
A0QZ33	MSMEG_3880, MSMEI_3790	Nitrilase/cyanide hydratase and apolipoprotein *N*-acyltransferase	0.130	17.500
A0R1H2	MSMEG_4752	Uncharacterized protein	0.062	3.333
A0R5K8	MSMEG_6227, MSMEI_6066	Transcriptional regulator, PadR family protein	0.057	8.000
A0R006	*wag31*, *ag84*, MSMEG_4217, MSMEI_4119	Cell wall synthesis protein Wag31 (antigen 84)	0.075	5.667
A0R2B0	MSMEG_5048, MSMEI_4921	Uncharacterized protein	0.035	5.500
A0QPX3	MSMEG_0550, MSMEI_0535	Sulfonate binding protein	0.085	3.500
A0QSG6	*rpsE*, MSMEG_1472, MSMEI_1436	30S ribosomal protein S5	0.075	12.333
A0R1B5	MSMEG_4692, MSMEI_4575	Uncharacterized protein MSMEG_4692/MSMEI_4575	0.082	4.500
A0QTM5	*pcaD*, MSMEG_1897, MSMEI_1857	3-Oxoadipate enol-lactonase (EC 3.1.1.24)	0.102	4.500
A0QU11	MSMEG_2037, MSMEI_1991	Bac_luciferase domain-containing protein	0.030	3.000
A0QS62	*rplJ*, MSMEG_1364, MSMEI_1325	50S ribosomal protein L10	0.078	8.500
A0QX20	*acnA*, *can*, MSMEG_3143, MSMEI_3062	Aconitate hydratase A (ACN) (aconitase) (EC 4.2.1.3)	0.055	14.500
A0QR90	MSMEG_1029, MSMEG_2309	Probable transcriptional regulatory protein	0.095	7.500
A0QT07	*sdhB*, MSMEG_1669	Succinate dehydrogenase, iron-sulfur protein (EC 1.3.99.1)	0.059	6.500
A0QS46	*rplA*, MSMEG_1347, MSMEI_1309	50S ribosomal protein L1	0.053	12.000
A0QZJ0	MSMEG_4042, MSMEI_3947	Transcriptional regulator, GntR family protein	0.050	6.000
A0QS97	*rpsG*, MSMEG_1399, MSMEI_1361	30S ribosomal protein S7	0.082	14.500
Q3L885	*pks*, MSMEG_0408, MSMEI_0398	Polyketide synthase (type I modular polyketide synthase)	0.023	43.000
A0QSG5	*rplR*, MSMEG_1471, MSMEI_1435	50S ribosomal protein L18	0.032	6.500
A0R616	MSMEG_6391, MSMEI_6223	Propionyl-CoA carboxylase beta chain (EC 6.4.1.3)	0.039	9.500

a The normalized score is the percentage of the total ion score of a single run attributable to a given protein. Normalized scores were averaged across replicates.

We additionally cross-referenced the 191 proteins observed across ClpC1^WT^ and ClpC1^EQ^ data sets with M. tuberculosis Clp protease interaction partners identified elsewhere by bacterial two-hybrid screening (23) or by LC-MS/MS upon knockdown of ClpP1 and ClpP2 (21). Thirty M. smegmatis proteins in our full-length ClpC1 interactome have M. tuberculosis homologs identified in these prior studies (see Table 3 below).

### Subcomponents of ClpC1 interact with specific cellular proteins.

The ClpC1 NTD is a discrete folded module thought to interact with substrates and adaptors, such as the N-end rule adaptor ClpS (22), but is dispensable for hexamer formation, ATP hydrolysis, and unfolding activities. We hypothesized that the NTD and the D1/D2 ATPase core of ClpC1 can bind to different interaction partners. To discriminate between interactions with the NTD and ClpC1 core, we created two truncated ClpC1 constructs: one consisting of only the NTD (ClpC1^NTD^) and another lacking the NTD but possessing the full D1 and D2 core (ClpC1^CORE^) (Fig. 1B). We repeated our pulldown and proteomics workflow with these constructs.

**FIG 4:**
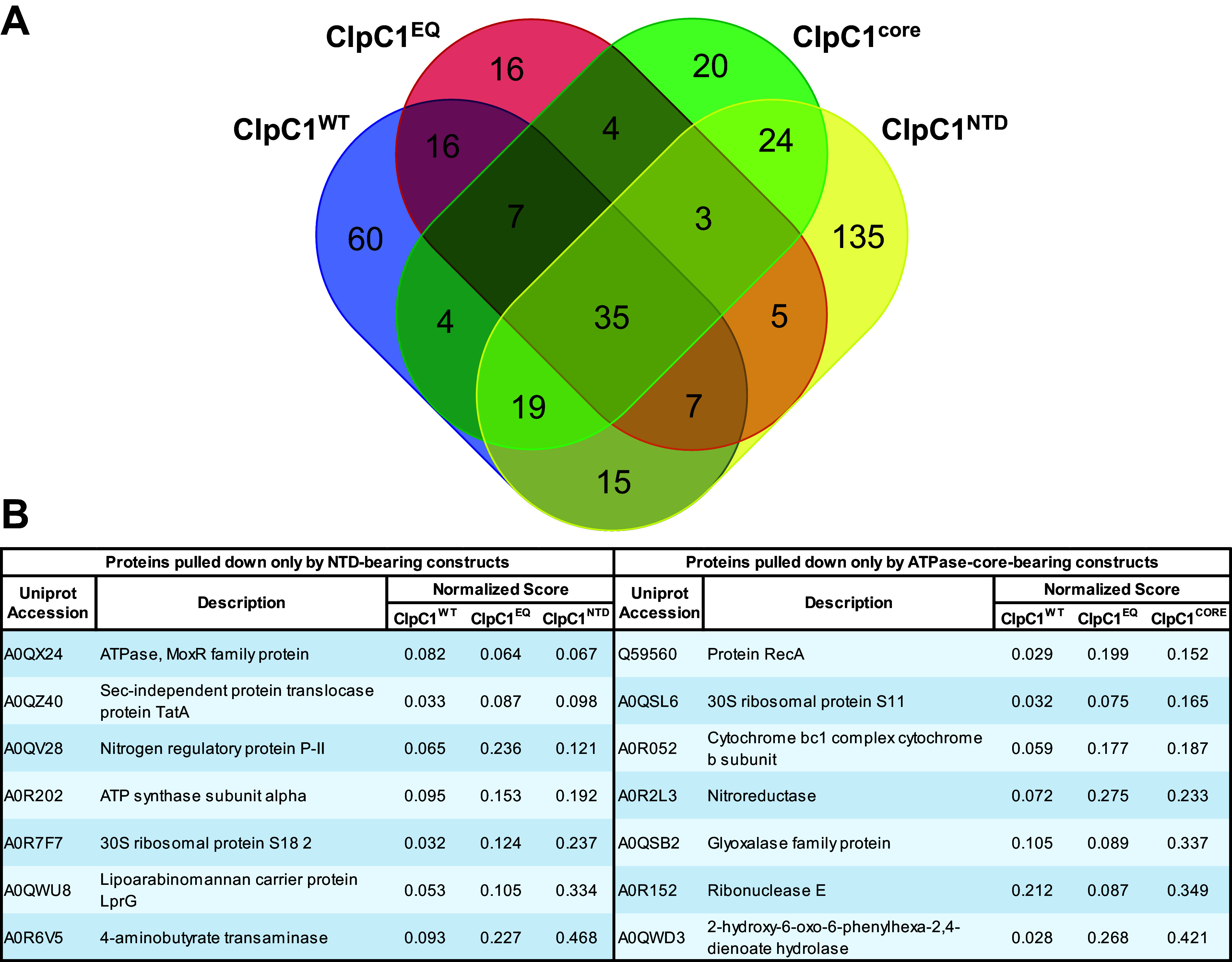
Comparative analysis of the ClpC1 interactome based on the different unfoldase components. (A) A Venn diagram shows protein targets observed to interact with one or more ClpC1 constructs. (B) The tabulated proteins were found to coimmunoprecipitate with all constructs bearing the amino-terminal domain (NTD) or the AAA+ ATPase core. Only 7 proteins were observed to interact with NTD alone. Similarly, as shown on the right only 7 proteins were observed interacting with the core-containing constructs and not in the NTD data sets.

We observed 243 proteins and 116 proteins that coimmunoprecipitate with ClpC1^NTD^ and ClpC1^CORE^, respectively (Tables S1 and S2). Comparing data sets, we found 42 proteins that interact with all core-bearing ClpC1 constructs (ClpC1^WT^, ClpC1^EQ^, and ClpC1^CORE^) (Fig. 4A). Notably, 72 out of 116 proteins (62%) that interact with the ClpC1^CORE^ were found in at least one other core-bearing data set, which provides additional confidence that these are bona fide interaction partners (Fig. 4A; Table S2). Of these, only 15 occurred exclusively in core-containing data sets, while the remaining 57 were also found in the ClpC1^NTD^ data set. Appearance in both ClpC1^NTD^ and ClpC1^CORE^ data sets may indicate that these proteins bind nonspecifically. Alternatively, they may genuinely interact with both the NTD and core, for which there is precedence in other organisms. For example, the Bacillus subtilis proteolytic adaptor MecA contacts both the ClpC NTD and M domain (63).

Forty-two proteins were found to interact with all NTD-bearing constructs (ClpC1^WT^, ClpC1^EQ^, and ClpC1^NTD^), although only 7 of these were exclusive to NTD-bearing constructs and absent from ClpC1^CORE^. Interestingly, only 84 proteins (35%) identified in the ClpC1^NTD^ data set were observed in at least one of the other NTD-bearing ClpC1^WT^ or ClpC1^EQ^ data sets, suggesting differences in how proteins interact with an isolated monomeric NTD and an NTD in the context of a ClpC1 hexamer.

Our analysis identified few proteins that consistently and uniquely interact with NTD-containing or core-containing ClpC1 constructs. Only 7 proteins coimmunoprecipitated with all of the NTD-bearing constructs and were absent from the ClpC1^CORE^ data set. Seven separate proteins interacted with all of the core-bearing constructs, but not ClpC1^NTD^ (Fig. 4B). Surprisingly, 35 proteins were found in all four data sets, suggesting either that some ClpC1-interacting proteins bind to both NTD and core regions or that truncated constructs are able to coassemble with endogenous ClpC1. Overall, our data suggest that many physiological ClpC1 interaction partners exist and that these may bind to multiple regions on the unfoldase.

### Mycobacterial ClpC1 has a diverse and far-reaching interactome.

**FIG 5:**
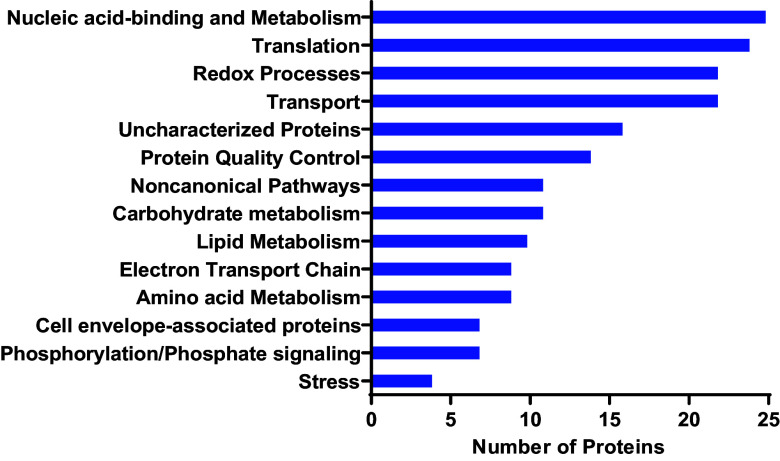
Full-length ClpC1 interacts with diverse groups of proteins in Mycolicibacterium smegmatis. Functional categorization of cellular proteins interacting with ClpC1^WT^ and/or ClpC1^EQ^ is shown. Also shown is the distribution of these proteins by Gene Ontology (GO) annotation based mostly on biological process. Functional classification was performed using Blast2GO and was based on annotation of the closest homologs.

To assess whether specific classes of proteins are preferentially targeted by M. smegmatis ClpC1, we performed Gene Ontology (GO) annotation analysis on the interactome using the Blast2GO software suite (64). GO terms were applied to M. smegmatis (strain ATCC 700084/MC^2^155) proteins based on the closest homologs present in the Swiss-Prot/UniProt database. We found that ClpC1 potentially regulates proteins with a diverse range of cellular functions (Fig. 5 and Table 2). Of particular interest were protein annotation groups that were highly represented in the full-length ClpC1 interactome. The most represented group of proteins were those associated with nucleic acid binding and metabolism, including transcriptional regulators (Hup, Rho, RpoC, Rne, RpoA, Pnp, IleS, RpoB, TopA, DnaX, SigA and transcriptional regulators belonging to the MarR, PadR, GntR, XRE, Crp/Fnr, TetR families, among other proteins). Additionally, proteins involved in redox reactions were identified, such as GabD2, IspG, Pks, DapB, among others. Another well-represented group were proteins involved in cellular transport (including SecA1, SecF, Ffh, LprG, TatA). Some of these proteins are transmembrane, but have cytoplasmic regions through which proteolytic regulation might occur (65, 66). In addition to nucleic acid metabolism, observed proteins were involved in other forms of metabolism, such as carbohydrate metabolism (GlmS, Eno, SucB, GlpK, SdhA, SdhB, AcnA, Mqo, Kgd), electron transport chain (Ndh, AtpA, AtpC, AtpD, AtpFH), amino acid metabolism (HemL, SerA, GabT, HisG, CarB, GlnA, MSMEG_3973, MSMEG_0688, MSMEI_3879), and lipid metabolism (fatty acid synthase MSMEG_4757, AcpM, acyl coenzyme A [acyl-CoA] synthases MSMEG_0599 and MSMEG_3767). Taken together, these observations suggest that ClpC1 plays a central role in the framework of mycobacterial cellular physiology by potentially regulating metabolic processes and/or ensuring quality control of metabolic enzymes (31).

**TABLE 2:** Proteins that interact with full-length ClpC1 (ClpC1^WT^ or ClpC1^EQ^)

UniProt accession no.	Gene name(s)	Protein description	Normalized score (mean ± SD)	
			ClpC1^WT^	ClpC1^EQ^
Amino acid metabolism				
A0QR33	*hemL*, MSMEG_0969, MSMEI_0943	Glutamate-1-semialdehyde 2,1-aminomutase (GSA)	0.09 ± 0.02	
A0QUY2	*serA*, MSMEG_2378, MSMEI_2318	d-3-Phosphoglycerate dehydrogenase (EC 1.1.1.95)	0.48 ± 0.64	
A0R6V5	*gabT*, MSMEG_6685, MSMEI_6505	4-Aminobutyrate transaminase (EC 2.6.1.19)	0.09 ± 0.1	0.23 ± 0.23
A0QZX1	*hisG*, MSMEG_4180, MSMEI_4082	ATP phosphoribosyltransferase (ATP-PRT) (ATP-PRTase)	0.04 ± 0.03	
A0QWS5	*carB*, MSMEG_3047, MSMEI_2973	Carbamoyl-phosphate synthase large chain (EC 6.3.5.5)	0.03 ± 0.02	
A0R079	*glnA*, *glnA1*, MSMEG_4290, MSMEI_4189	Glutamine synthetase (GS) (EC 6.3.1.2)	0.16 ± 0.21	
A0QZC4	MSMEG_3973, MSMEI_3878	*N*-Methylhydantoinase	0.39 ± 0.19	0.7 ± 1.05
A0QQA8	MSMEG_0688, MSMEI_0671	Aspartate aminotransferase	0.29 ± 0.31	
I7G417	MSMEI_3879	5-Oxoprolinase (ATP-hydrolyzing) (EC 3.5.2.9)	0.32 ± 0.27	0.64 ± 0.53
Carbohydrate metabolism				
O68956	*glmS*, MSMEG_1568, MSMEI_1531	Glutamine-fructose-6-phosphate aminotransferase	0.05 ± 0.04	
A0R3B8	*eno*, MSMEG_5415, MSMEI_5267	Enolase (EC 4.2.1.11) (2-phospho-d-glycerate hydrolyase)	0.13 ± 0.06	
A0R364	MSMEG_5358, MSMEI_5212	Acetamidase/formamidase family protein	0.17 ± 0.16	0.06 ± 0.03
A0R266	MSMEG_5002, MSMEI_4876	AAA_27 domain-containing protein	0.06 ± 0.01	
A0R072	*sucB*, MSMEG_4283, MSMEI_4182	Dihydrolipoamide acetyltransferase component of PDH complex	0.1 ± 0.14	
A0R729	*glpK*, MSMEG_6759, MSMEI_6577	Glycerol kinase (EC 2.7.1.30)	0.17 ± 0.25	0.22 ± 0.13
A0QT08	*sdhA*, MSMEG_1670, MSMEI_1630	Succinate dehydrogenase flavoprotein subunit (EC 1.3.5.1)	0.12 ± 0.06	0.06 ± 0.05
A0QT07	*sdhB*, MSMEG_1669, MSMEI_1629	Succinate dehydrogenase, iron-sulfur protein (EC 1.3.99.1)		0.06 ± 0.08
A0QX20	*acnA*, *can*, MSMEG_3143, MSMEI_3062	Aconitate hydratase A (ACN) (aconitase) (EC 4.2.1.3)		0.06 ± 0.08
A0QVL2	*mqo*, MSMEG_2613, MSMEI_2551	Probable malate:quinone oxidoreductase	0.36 ± 0.16	
A0R2B1	*kgd*, *sucA*, MSMEG_5049, MSMEI_4922	Multifunctional 2-oxoglutarate metabolism enzyme	0.12 ± 0.12	
Cell envelope-associated proteins				
A0QTT7	MSMEG_1959, MSMEI_1915	UPF0182 protein MSMEG_1959/MSMEI_1915	0.14 ± 0.1	
A0QP93	MSMEG_0317	Uncharacterized protein	0.19 ± 0.21	
A0QYF6	MSMEG_3641, MSMEI_3556	VWFA domain-containing protein	0.04 ± 0.02	
A0QWT6	MSMEG_3058, MSMEI_2982	Lipoprotein		0.14 ± 0.06
A0R1H2	MSMEG_4752	Uncharacterized protein		0.06 ± 0.06
A0R006	*wag31*, *ag84*, MSMEG_4217, MSMEI_4119	Cell wall synthesis protein Wag31 (antigen 84)		0.07 ± 0.04
A0R5I3	MSMEG_6201	Transglycosylase	0.04 ± 0.01	
Electron transport chain				
A0QYD6	*ndh*, MSMEG_3621, MSMEI_3536	NADH dehydrogenase (EC 1.6.99.3)	0.09 ± 0.12	
A0R052	MSMEG_4263, MSMEI_4163	Ubiquinol-cytochrome *c* reductase cytochrome *b* subunit	0.06 ± 0.03	0.18 ± 0.02
A0R057	*ctaC*, MSMEG_4268, MSMEI_4167	Cytochrome *c* oxidase subunit 2 (EC 1.9.3.1)	0.07 ± 0.01	0.21 ± 0.2
A0R203	*atpFH*, *atpF*, *atpH*, MSMEG_4939, MSMEI_4812	ATP synthase subunit b-delta	0.59 ± 0.42	0.55 ± 0.31
A0R200	*atpD*, MSMEG_4936, MSMEI_4809	ATP synthase subunit beta	0.19 ± 0.07	0.12 ± 0.04
A0R202	*atpA*, MSMEG_4938, MSMEI_4811	ATP synthase subunit alpha	0.1 ± 0.04	0.15 ± 0.06
A0R051	*qcrA*, MSMEG_4262, MSMEI_4162	Ubiquinol-cytochrome *c* reductase iron-sulfur subunit	0.14 ± 0.07	
A0QQB0	MSMEG_0690, MSMEI_0673	Iron-sulfur cluster-binding protein	0.09 ± 0.06	
A0R1Z9	*atpC*, MSMEG_4935, MSMEI_4808	ATP synthase epsilon chain		0.06 ± 0.04
Lipid metabolism				
A0QPE8	*fadA2*, MSMEG_0373, MSMEI_0366	3-Ketoacyl-CoA thiolase (EC 2.3.1.16)	0.06 ± 0.04	
A0R1H7	MSMEG_4757, MSMEI_4637	Fatty acid synthase	0.4 ± 0.39	
A0R5R5	MSMEG_6284, MSMEI_6119	Cyclopropane-fatty-acyl-phospholipid synthase	0.05 ± 0.03	0.07 ± 0.02
A0R2E8	*fadD6*, MSMEG_5086, MSMEI_4960	Very-long-chain acyl-CoA synthetase (EC 6.2.1.−) (EC 6.2.1.3)	0.09 ± 0.08	
A0R0B3	*acpM*, MSMEG_4326, MSMEI_4226	Meromycolate extension acyl carrier protein (ACP)	0.07 ± 0.08	0.11 ± 0.02
A0R042	MSMEG_4254, MSMEI_4153	AMP-binding enzyme	0.03 ± 0.03	
A0QYS3	MSMEG_3767, MSMEI_3678	Acyl-CoA synthase	0.07 ± 0.09	
A0R616	*accD4*, MSMEG_6391, MSMEI_6223	Propionyl-CoA carboxylase beta chain (EC 6.4.1.3)		0.04 ± 0.05
A0QQ22	*fadD2*, MSMEG_0599, MSMEI_0583	Acyl-CoA synthase	0.01 ± 0.02	
A0R0B4	*kasA*, MSMEG_4327, MSMEI_4227	3-Oxoacyl-[acyl-carrier-protein] synthase 1 (EC 2.3.1.41)	0.06 ± 0.06	
Noncanonical pathways				
A0R5C0	MSMEG_6137, MSMEI_5978	Nonribosomal peptide synthetase	0.01 ± 0	
A0QPH5	MSMEG_0400	Peptide synthetase	0.11 ± 0.11	
A0QY29	*eis*, MSMEG_3513, MSMEI_3433	Uncharacterized *N*-acetyltransferase MSMEG_3513 (EC 2.3.1.−)	0.05 ± 0.04	
A0QV52	MSMEG_2450, MSMEI_2388	Adenosylmethionine-8-amino-7-oxononanoate transaminase	0.03 ± 0.02	
A0QWD3	MSMEG_2900, MSMEI_2827	2-Hydroxy-6-oxo-6-phenylhexa-2,4-dienoate hydrolase (EC 3.7.1.−)	0.03 ± 0.02	0.27 ± 0.24
A0QZ01	*lipT*, MSMEG_3848, MSMEI_3758	Carboxylic ester hydrolase (EC 3.1.1.−)	0.02 ± 0.02	
A0QZ33	MSMEG_3880, MSMEI_3790	Nitrilase/cyanide hydratase		0.13 ± 0.07
A0QTM5	*pcaD*, MSMEG_1897, MSMEI_1857	3-Oxoadipate enol-lactonase (EC 3.1.1.24)		0.1 ± 0.05
A0R617	*pks13*, MSMEG_6392, MSMEI_6224	Polyketide synthase	0.16 ± 0.14	
A0QTE1	*accA3*, MSMEG_1807, MSMEI_1762	Acetyl/propionyl-coenzyme A carboxylase alpha chain	0.13 ± 0.17	
A0QV28	*glnB*, MSMEG_2426, MSMEI_2365	Nitrogen regulatory protein P-II	0.06 ± 0.08	0.24 ± 0.16
Nucleic acid binding and metabolism, including transcriptional regulators				
A0QTQ8	*rhlE*, MSMEG_1930, MSMEI_1889	DEAD/DEAH box helicase	0.19 ± 0.09	0.16 ± 0.07
A0R7J3	*parB*, MSMEG_6938, MSMEI_6746	ParB-like partition protein	0.1 ± 0.09	
A0QT91	*lhr*, MSMEG_1757, MSMEI_1715	DEAD/DEAH box helicase	0.07 ± 0.02	
Q9ZHC5	*hup*, *hlp*, MSMEG_2389, MSMEI_2329	DNA-binding protein HU homolog (histone-like protein) (Hlp)	0.14 ± 0.16	0.18 ± 0
A0R656	MSMEG_6431, MSMEI_6263	HTH cro/C1-type domain-containing protein	0.01 ± 0.01	
A0QYG0	MSMEG_3645, MSMEI_3559	BFN domain-containing protein	0.06 ± 0.0009	
A0R218	*rho*, MSMEG_4954, MSMEI_4827	Transcription termination factor Rho (EC 3.6.4.−)	0.6 ± 0.5	0.54 ± 0.02
A0QS66	*rpoC*, MSMEG_1368, MSMEI_1329	DNA-directed RNA polymerase subunit beta′	0.45 ± 0.57	0.36 ± 0.29
A0R152	*rne*, MSMEG_4626, MSMEI_4509	Ribonuclease E (RNase E) (EC 3.1.26.12)	0.21 ± 0.18	0.09 ± 0.06
A0QSL8	*rpoA*, MSMEG_1524, MSMEI_1488	DNA-directed RNA polymerase subunit alpha	0.05 ± 0.02	
A0QVQ5	*pnp*, *gpsI*, MSMEG_2656, MSMEI_2593	Polyribonucleotide nucleotidyltransferase	0.03 ± 0.05	
A0QX46	*ileS*, MSMEG_3169, MSMEI_3087	Isoleucine-tRNA ligase (EC 6.1.1.5) (isoleucyl-tRNA synthetase)	0.02 ± 0.02	
P60281	*rpoB*, MSMEG_1367, MSMEI_1328	DNA-directed RNA polymerase subunit beta	0.24 ± 0.29	
A0R5D9	*topA*, MSMEG_6157, MSMEI_5999	DNA topoisomerase 1 (EC 5.6.2.1) (DNA topoisomerase I)	0.15 ± 0.19	
A0QW71	MSMEG_2839, MSMEI_2765	Transcriptional accessory protein	0.05 ± 0.04	
A0R5R6	*dnaX*, MSMEG_6285, MSMEI_6120	DNA polymerase III subunit gamma/tau (EC 2.7.7.7)	0.05 ± 0.02	
A0R3L1	MSMEG_5512	Magnesium chelatase	0.32 ± 0.13	0.5 ± 0.6
A0QVD7	MSMEG_2538, MSMEI_2478	MarR-family protein transcriptional regulator	0.03 ± 0.01	0.08 ± 0.01
A0R5K8	MSMEG_6227, MSMEI_6066	Transcriptional regulator, PadR family protein		0.06 ± 0.05
A0QR90	MSMEG_1029, MSMEG_2309	Probable transcriptional regulatory protein		0.09 ± 0.13
A0QZJ0	MSMEG_4042, MSMEI_3947	Transcriptional regulator, GntR family protein		0.05 ± 0.07
A0QW02	*sigA*, *rpoD*, MSMEG_2758, MSMEI_2690	RNA polymerase sigma factor SigA	0.11 ± 0.05	
A0R5H1	MSMEG_6189, MSMEI_6029	Transcriptional regulator, Crp/Fnr family protein	0.08 ± 0.03	0.27 ± 0.17
A0QQY3	MSMEG_0918, MSMEI_0896	Transcriptional regulator, XRE family protein	0.02 ± 0.01	
A0QR26	MSMEG_0962, MSMEI_0936	TetR family protein transcriptional regulator	0.01 ± 0.01	
Phosphorylation/phosphate signaling				
A0R5N8	*ask*, MSMEG_6257, MSMEI_6095	Aspartokinase (EC 2.7.2.4)	0.2 ± 0.23	0.09 ± 0.04
A0QU86	MSMEG_2116, MSMEI_2068	PTS system, glucose-specific IIBC component	0.03 ± 0.04	
A0QNG5	*ppp*, MSMEG_0033, MSMEI_0035	Protein phosphatase 2C	0.02 ± 0	
A0QNG2	*pknA*, MSMEG_0030, MSMEI_0032	Serine/threonine protein kinase PknA (EC 2.7.1.−)	0.01 ± 0.01	
A0QV12	MSMEG_2410, MSMEI_2349	Putative serine-threonine protein kinase	0.05 ± 0.01	0.16 ± 0.12
A0R0T1	MSMEG_4497, MSMEI_4386	PhoH family protein	0.1 ± 0.12	0.16 ± 0.14
A0QX24	*moxR*, MSMEG_3147	ATPase, MoxR family protein	0.08 ± 0.06	0.06 ± 0.09
Protein quality control				
A0QQU5	*groEL2*, *groL2*, MSMEG_0880, MSMEI_0859	60-kDa chaperonin 1 (GroEL protein 1) (protein Cpn60 1)	1.68 ± 1.5	2.41 ± 0.76
A0QSS4	*groEL1*, *groL1*, MSMEG_1583, MSMEI_1545	60-kDa chaperonin 2 (GroEL protein 2) (protein Cpn60 2)	1.04 ± 0.77	0.99 ± 0.13
A0QW35	MSMEG_2792, MSMEI_2723	Clp amino-terminal domain protein	0.09 ± 0.05	0.29 ± 0.1
A0R085	MSMEG_4296, MSMEI_4195	Protease	0.05 ± 0	
A0QSH0	*sppA*, MSMEG_1476, MSMEI_1440	Signal peptide peptidase SppA, 67K type (EC 3.4.−.−)	0.06 ± 0.04	0.14 ± 0.05
A0QQM2	MSMEG_0806, MSMEI_0787	Hydrolase	0.05 ± 0.04	
A0R196	*clpX*, MSMEG_4671, MSMEI_4553	ATP-dependent Clp protease ATP-binding subunit ClpX	0.03 ± 0.01	
A0QTT5	MSMEG_1957, MSMEI_1913	Uncharacterized protein	0.09 ± 0.11	0.09 ± 0.01
A0R6J9	*est*, MSMEG_6575, MSMEI_6397	Beta-lactamase	0.04 ± 0.03	
A0QQD0	*dnaJ*, *dnaJ1*, MSMEG_0711, MSMEI_0694	Chaperone protein DnaJ1	0.53 ± 0.45	0.83 ± 0.94
A0R0T8	*dnaJ*, *dnaJ2*, MSMEG_4504, MSMEI_4392	Chaperone protein DnaJ2	0.09 ± 0.01	
A0QQC8	*dnaK*, MSMEG_0709, MSMEI_0692	Chaperone protein DnaK (HSP70)	0.25 ± 0.3	
A0R199	*tig*, MSMEG_4674, MSMEI_4557	Trigger factor (TF) (EC 5.2.1.8) (PPIase)	0.16 ± 0.21	
A0QWK5	*ppiB*, MSMEG_2974, MSMEI_2900	Peptidyl-prolyl *cis*-*trans* isomerase (PPIase) (EC 5.2.1.8)		0.09 ± 0
Redox processes				
A0QPE7	*fabG4*, MSMEG_0372, MSMEI_0365	Oxidoreductase, short-chain dehydrogenase/reductase family protein	0.35 ± 0.25	0.33 ± 0.04
A0QR89	MSMEG_1028, MSMEG_2308	Geranylgeranyl reductase	0.25 ± 0.25	0.25 ± 0.22
A0R696	MSMEG_6471, MSMEI_6299	Glycine/d-amino acid oxidase	0.05 ± 0.01	
A0QR91	MSMEG_1030, MSMEG_2310	Monooxygenase	0.12 ± 0.09	
A0R4Q0	*gabD2*, MSMEG_5912, MSMEI_5752	Putative succinate-semialdehyde dehydrogenase	0.01 ± 0	
A0R730	*glpD2*, MSMEG_6761, MSMEI_6578	Glycerol-3-phosphate dehydrogenase (EC 1.1.5.3)	0.09 ± 0.11	
A0R226	MSMEG_4962, MSMEI_4836	RemO protein	0.02 ± 0.02	
A0QSL0	MSMEG_1516, MSMEI_1480	Thioredoxin reductase	0.01 ± 0	
A0QVH9	*ispG*, MSMEG_2580, MSMEI_2518	4-Hydroxy-3-methylbut-2-en-1-yl diphosphate synthase (flavodoxin)	0.01 ± 0.01	
A0QSB2	MSMEG_1417, MSMEI_1382	Glyoxalase family protein	0.11 ± 0.12	0.09 ± 0.01
A0QQV4	*gabD2*, MSMEG_0889, MSMEI_0868	Aldehyde dehydrogenase	0.14 ± 0	0.11 ± 0.03
A0R2L3	MSMEG_5155, MSMEI_5022	Nitroreductase	0.07 ± 0.04	0.28 ± 0.07
A0QYH8	MSMEG_3663, MSMEI_3576	Oxidoreductase	0.06 ± 0.01	
A0QSB9	*mftD*, *lldD1*, MSMEG_1424, MSMEI_1389	FMN-dependent dehydrogenase	0.02 ± 0.03	
A0R461	MSMEG_5715, MSMEI_5564	Bac_luciferase domain-containing protein		0.39 ± 0.31
A0QRD2	MSMEG_1073, MSMEI_1041	Oxidoreductase, short-chain dehydrogenase/reductase family protein		0.09 ± 0.05
A0QU11	MSMEG_2037, MSMEI_1991	Bac_luciferase domain-containing protein		0.03 ± 0.04
Q3L885	*pks*, MSMEG_0408, MSMEI_0398	Polyketide synthase (type I modular polyketide synthase)		0.02 ± 0.03
A0QXA1	*gltB*, MSMEG_3225, MSMEI_3143	Ferredoxin-dependent glutamate synthase 1 (EC 1.4.7.1)	0.03 ± 0.01	
A0QXI7	*dapB*, MSMEG_3317, MSMEI_3231	Dihydrodipicolinate reductase, N-terminal domain protein	0.06 ± 0.06	
A0R221	MSMEG_4957, MSMEI_4830	Homoserine dehydrogenase (EC 1.1.1.3)	0.24 ± 0.18	0.2 ± 0.09
A0R0W1	MSMEG_4527, MSMEI_4414	Ferredoxin sulfite reductase (EC 1.8.7.1)	0.03 ± 0.02	
Stress response				
A0QQQ1	*sodC*, MSMEG_0835, MSMEI_0816	Superoxide dismutase [Cu-Zn] (EC 1.15.1.1)	0.1 ± 0.02	
A0QS28	*recD*, MSMEG_1325, MSMEI_1288	RecBCD enzyme subunit RecD (EC 3.1.11.5)	0.02 ± 0.01	
A0QUM3	*cstA*, MSMEG_2259, MSMEI_2203	Carbon starvation protein A	0.02 ± 0.02	
Q59560	*recA*, MSMEG_2723, MSMEI_2656	Protein RecA (recombinase A)	0.03 ± 0.04	0.2 ± 0.03
Translation				
A0QS98	*tuf*, MSMEG_1401, MSMEI_1363	Elongation factor Tu (EF-Tu)	0.24 ± 0.18	0.33 ± 0.28
A0QVM7	*infB*, MSMEG_2628, MSMEI_2565	Translation initiation factor IF-2	0.46 ± 0.46	
A0QYY6	*rpsA*, MSMEG_3833, MSMEI_3743, LJ00_19040	30S ribosomal protein S1	0.16 ± 0.2	
A0R7F7	*rpsR2*, *rpsR1*, MSMEG_6895, MSMEI_6711	30S ribosomal protein S18 2	0.03 ± 0.01	0.12 ± 0.11
A0QSE0	*rpsQ*, MSMEG_1445, MSMEI_1409	30S ribosomal protein S17	0.02 ± 0.01	
A0QSD7	*rpsC*, MSMEG_1442, MSMEI_1406	30S ribosomal protein S3	0.23 ± 0.19	0.36 ± 0.32
A0QV03	*rpmB*, *rpmB-3*, MSMEG_2400, MSMEI_2340	50S ribosomal protein L28	0.02 ± 0.01	
A0QSL6	*rpsK*, MSMEG_1522, MSMEI_1486	30S ribosomal protein S11	0.03 ± 0.03	0.07 ± 0.07
A0QSD1	*rplC*, MSMEG_1436, MSMEI_1400	50S ribosomal protein L3	0.1 ± 0.14	0.25 ± 0.09
A0R3I9	*rpmF*, MSMEG_5489, MSMEI_5337	50S ribosomal protein L32	0.03 ± 0.03	0.15 ± 0.02
A0QSL5	*rpsM*, MSMEG_1521, MSMEI_1485	30S ribosomal protein S13	0.04 ± 0.04	
A0R150	*rpmA*, MSMEG_4624, MSMEI_4507	50S ribosomal protein L27	0.02 ± 0.02	
A0QVB9	*tsf*, MSMEG_2520, MSMEI_2461	Elongation factor Ts (EF-Ts)	0.03 ± 0.04	
A0QSD0	*rpsJ*, MSMEG_1435, MSMEI_1399	30S ribosomal protein S10	0.11 ± 0.02	0.17 ± 0.24
A0QSD6	*rplV*, MSMEG_1441, MSMEI_1405	50S ribosomal protein L22	0.03 ± 0.01	
A0QSG0	*rplX*, MSMEG_1466, MSMEI_1430	50S ribosomal protein L24	0.02 ± 0.02	
A0QYU7	*rpmI*, MSMEG_3792, MSMEI_3704	50S ribosomal protein L35	0.01 ± 0.01	
A0QSP8	*rplM*, MSMEG_1556, MSMEI_1519	50S ribosomal protein L13		0.08 ± 0.07
A0QSD2	*rplD*, MSMEG_1437, MSMEI_1401	50S ribosomal protein L4		0.05 ± 0.07
A0QSG6	*rpsE*, MSMEG_1472, MSMEI_1436	30S ribosomal protein S5		0.07 ± 0.03
A0QS62	*rplJ*, MSMEG_1364, MSMEI_1325	50S ribosomal protein L10		0.08 ± 0.03
A0QS46	*rplA*, MSMEG_1347, MSMEI_1309	50S ribosomal protein L1		0.05 ± 0.07
A0QS97	*rpsG*, MSMEG_1399, MSMEI_1361	30S ribosomal protein S7		0.08 ± 0.03
A0QSG5	*rplR*, MSMEG_1471, MSMEI_1435	50S ribosomal protein L18		0.03 ± 0.04
Transport				
A0QQ65	MSMEG_0643, MSMEI_0627	Extracellular solute-binding protein, family protein 5, putative	0.56 ± 0.25	0.63 ± 0.32
A0QXC0	MSMEG_3247, MSMEI_3164	Branched-chain amino acid ABC transporter substrate-binding protein	0.57 ± 0.34	0.41 ± 0.18
A0QWL3	MSMEG_2982, MSMEI_2907	Putative periplasmic binding protein	0.46 ± 0.3	0.44 ± 0.04
P71533	*secA1*, MSMEG_1881, MSMEI_1840	Protein translocase subunit SecA 1	0.07 ± 0.06	0.04 ± 0.05
A0QXF3	MSMEG_3280, MSMEI_3196	Polyamine-binding lipoprotein	0.06 ± 0.03	
A0QRB1	MSMEG_1052, MSMEG_2332	Amino acid carrier protein	0.13 ± 0.13	0.1 ± 0.07
A0QYK4	MSMEG_3689, MSMEI_3602	Sodium:solute symporter	0.05 ± 0.03	0.1 ± 0.03
A0R261	MSMEG_4999, MSMEI_4871	Bacterial extracellular solute-binding protein, family protein 5	0.05 ± 0.03	
A0QWJ3	*secF*, MSMEG_2962, MSMEI_2888	Protein-export membrane protein SecF	0.02 ± 0	
A0R5T7	MSMEG_6307, MSMEI_6142	Glutamine-binding periplasmic protein	0.05 ± 0.06	
A0R2C0	*sugC*, MSMEG_5058, MSMEI_4931	ABC transporter, ATP-binding protein SugC	0.02 ± 0.01	
A0QT21	MSMEG_1683, MSMEI_1642	Cytosine/purine/uracil/thiamine/allantoin permease family protein	0.02 ± 0.02	
A0QVX3	MSMEG_2727, MSMEI_2660	Glutamate-binding protein	0.22 ± 0.03	0.39 ± 0.03
A0QXB0	MSMEG_3235, MSMEI_3153	ABC-type amino acid transport system, secreted component	0.23 ± 0.09	0.24 ± 0.2
A0R0W7	MSMEG_4533, MSMEI_4420	Sulfate-binding protein	0.16 ± 0.01	0.34 ± 0.23
A0QV32	*ffh*, MSMEG_2430, MSMEI_2369	Signal recognition particle protein (fifty-four homolog)	0.13 ± 0.05	
A0QWU8	*lprG*, MSMEG_3070, MSMEI_2993	Lipoarabinomannan carrier protein LprG	0.05 ± 0.02	0.1 ± 0.02
A0QSV2	MSMEG_1612, MSMEI_1573	Extracellular solute-binding protein, family protein 3	0.09 ± 0.05	0.11 ± 0.02
A0QZ40	*tatA*, MSMEG_3887, MSMEI_3797	Sec-independent protein translocase protein TatA	0.03 ± 0	0.09 ± 0.01
A0QPX3	MSMEG_0550, MSMEI_0535	Sulfonate binding protein		0.09 ± 0.02
A0QNN8	*pntA*, MSMEG_0110, MSMEI_0106	NAD(P) transhydrogenase, alpha subunit (EC 1.6.1.1)	0.06 ± 0.01	
A0QSY1	MSMEG_1642, MSMEI_1603	ABC transporter, ATP-binding protein	0.08 ± 0.09	
Uncharacterized proteins				
A0R3L0	MSMEG_5511, MSMEI_5359	von Willebrand factor, type A	0.05 ± 0	0.08 ± 0.02
A0QPE3	MSMEG_0368, MSMEI_0361	Uncharacterized protein	0.03 ± 0.01	
A0R576	*lsr2*, MSMEG_6092, MSMEI_5934	Lsr2 protein	0.05 ± 0.03	0.15 ± 0.21
A0QNZ9	MSMEG_0222, MSMEI_0215	DUF2786 domain-containing protein	0.04 ± 0.03	
I7FPJ2	MSMEI_4181	Uncharacterized protein	0.01 ± 0.01	
A0R6E9	MSMEG_6524, MSMEI_6350	ABC polyamine/opine/phosphonate transporter	0.07 ± 0.09	
Q3I5Q7	MSMEG_0919, MSMEI_0897	HBHA-like protein (heparin-binding hemagglutinin)	0.14 ± 0.13	0.25 ± 0.21
A0QTG7	MSMEG_1835, MSMEI_1793	TobH protein	0.22 ± 0.07	0.31 ± 0.15
A0QYR3	MSMEG_3754, MSMEI_3665	TPR-repeat-containing protein	0.13 ± 0.02	0.07 ± 0.04
A0QZY3	MSMEG_4192, MSMEI_4094	Uncharacterized protein	0.07 ± 0.05	0.05 ± 0.08
A0R562	MSMEG_6078, MSMEI_5918	LpqE protein	0.04 ± 0.03	
A0R6C7	MSMEG_6502, MSMEI_6330	Uncharacterized protein	0.04 ± 0	
A0R2T1	MSMEG_5223	Uncharacterized protein	0.04 ± 0.01	
A0QVU2	MSMEG_2695, MSMEI_2629	35-kDa protein		0.08 ± 0
A0R2B0	MSMEG_5048, MSMEI_4921	Uncharacterized protein		0.03 ± 0.05
A0R1B5	MSMEG_4692, MSMEI_4575	Uncharacterized protein MSMEG_4692/MSMEI_4575		0.08 ± 0.02

Proteins involved in protein quality control, such as protein folding and unfolding, proteome turnover, and protein homeostasis in general, were also important constituents of the interactome (Fig. 5 and Table 2). These included chaperone proteins GroL1, GroL2, DnaJ1, DnaJ2, DnaK, and Tig and Clp proteins ClpX and ClpC2 (MSMEG_2792). ClpC1 is thus named because of the existence of the orthologous ClpC2 which possesses homology to the ClpC1 N-terminal domain, but lacks AAA+ ATPase modules (22, 67). As ClpC1 is itself involved in protein homeostasis and protein fate in general, it is not surprising to see its interaction with these proteins. Also, as noted above, components of the ribosome and other proteins involved in translation were abundant as well, making up ~13% of the total data set (Fig. 5). It is possible that nascent ClpC1 stays in contact with the ribosome during folding, either directly or indirectly through other elements of folding machinery. Alternatively, ribosomal proteins are commonly observed as contaminants in cell-based mass spectrometry experiments (68).

It is also important to point out that for the proteins specifically interacting with either ClpC1 NTD or core alone (Fig. 4), there was some functional diversity. For the NTD-interacting proteins, there were proteins involved in transport (TatA and LprG), phosphorylation/dephosphorylation (MoxR), the electron transport chain (AtpA), amino acid metabolism (GabT), noncanonical pathways (MSMEG_2426), and translation (RpsR2). The ClpC1 core-interacting proteins had roles in redox processes (MSMEG_5155 and MSMEG_1417), stress response (RecA), translation (RpsK), the electron transport chain (MSMEG_4263), nucleic acid binding and metabolism (Rne), and noncanonical pathways (MSMEG _2900).

Taken together, we observe a functionally diverse set of cellular proteins that interact with M. smegmatis ClpC1, revealing a broad diversity of putative substrates and interaction partners of the ClpC1P1P2 protease. This buttresses just how far-reaching its regulatory effects on cellular proteins could be and may help account for the essentiality of these proteases in mycobacteria (19, 27–31).

### Physiochemical analysis of termini of interacting proteins.

Some Clp protease substrates are recognized by short terminal degron sequences of various lengths and compositions. M. smegmatis ClpC1 recognizes model substrates bearing a C-terminal SsrA sequence (ADSNQRDYALAA) (13). M. tuberculosis ClpC1 recognizes Hsp20 via the C-terminal sequence TQAQRIAITK (21) and PanD via the C-terminal sequence NAGELLDPRLGVG (20). In addition, substrates displaying some hydrophobic N-terminal residues are delivered to ClpC1 by the adaptor ClpS as part of the N-end rule proteolytic pathway (23, 69).

**FIG 6:**
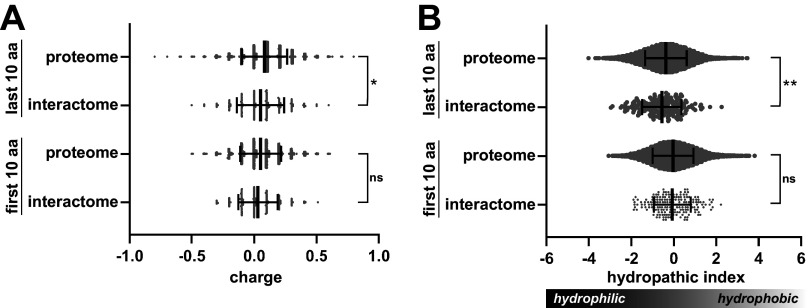
Physicochemical analysis of terminal sequences of full-length ClpC1-interacting proteins. (A) Violin plots illustrate the average charges of the first (N-terminal) and last (C-terminal) amino acids in the ClpC1 interactome and in the entire M. smegmatis proteome. Central lines indicate median values, with error bars indicating 1 SD. (B) Mean hydrophobicity of terminal residues in the ClpC1 interactome and control data sets is shown. For both metrics, the values plotted are the average residue value per terminus. Significance was assessed by two-tailed Welch’s *t* test. *, *P* < 0.05; **, *P* < 0.01; ns, not significant.

Because the sequence determinants required for recognition by ClpC1 are not well defined, we compared terminal sequences of proteins pulled down by full-length ClpC1 (ClpC1^WT^ or ClpC1^EQ^) to the terminal sequences found across the entire M. smegmatis proteome (Fig. 6). The average charge and hydrophobicity of the first and last 10 amino acids (aa) of protein sequences varied widely. We saw no statistically significant differences in these parameters for residues at the N terminus. However, C-terminal regions of interactome proteins were, on average, less positively charged (closer to neutral) and more hydrophilic than equivalent regions from the full proteome. These differences were statistically significant, although the magnitude of the shift was much smaller than the standard deviation (SD) in either data set. The apparent preference for hydrophilic C-terminal character is surprising, as ATP-dependent proteases are often thought to recognize exposed hydrophobic regions as markers for protein misfolding (8). This analysis suggests either a slight preference for uncharged polar termini or that polar termini are more exposed to solvent and more available for ClpC1 binding. Additionally, the discrepancy between expected and observed physicochemical parameters might be explained by a preponderance of ClpC1-interacting proteins that are not proteolytic substrates, and thus may not be subject to the same recognition trends as the substrates.

### ClpC1P1P2 recognizes MSMEI_3879 as a proteolytic substrate.

**FIG 7:**
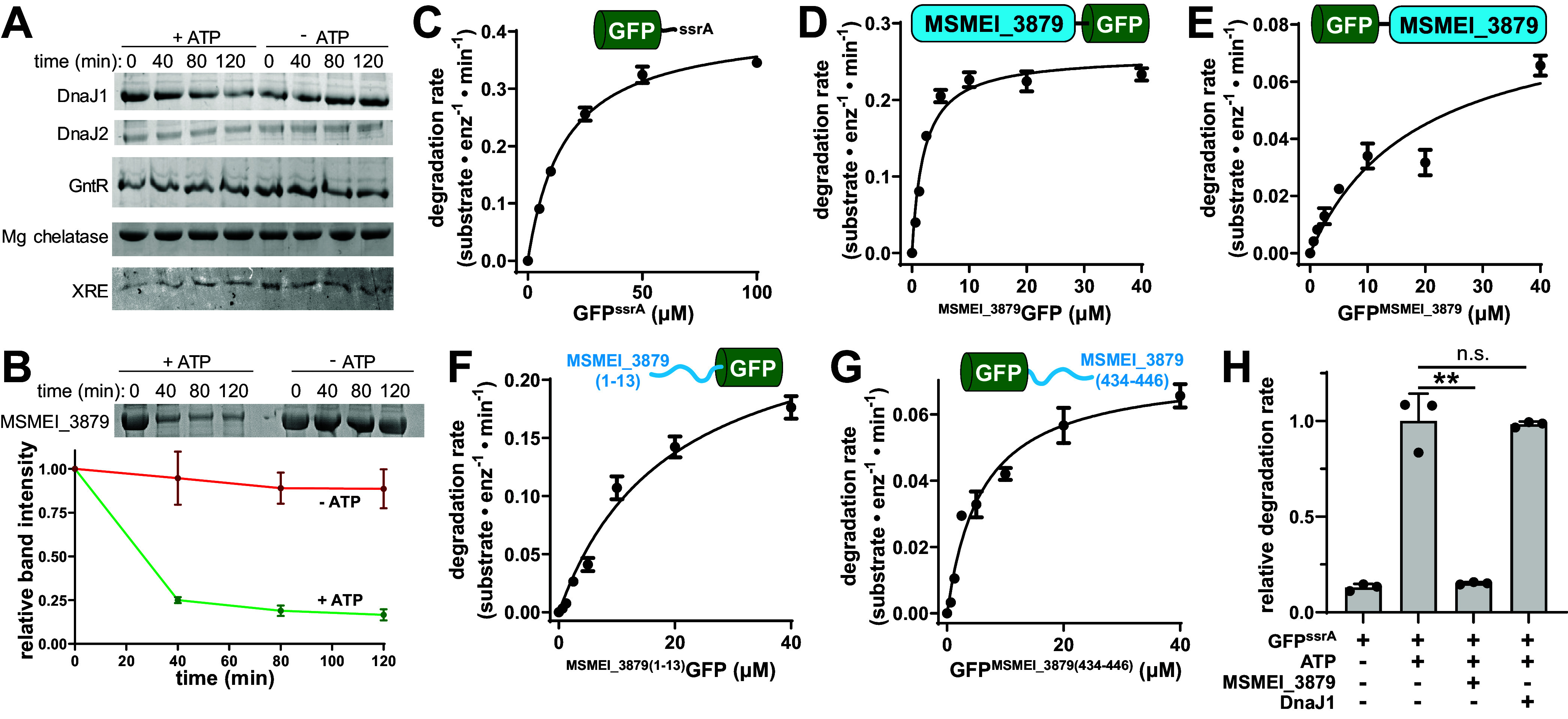
MSMEI_3879 is a substrate of M. smegmatis ClpC1. (A) *In vitro* degradation assays of DnaJ1, DnaJ2, GntR, magnesium chelatase, and XRE by 1 μM ClpC1 and 1 μM ClpP1P2, monitored by SDS-PAGE. (B) ATP-dependent degradation of 10 μM MSMEI_3879 by ClpC1P1P2 was observed *in vitro* by SDS-PAGE, with gel densitometry of three replicate assays shown. (C) Michaelis-Menten analysis of GFP^ssrA^ proteolysis by 1 μM ClpC1P1P2, as a function of substrate concentration, reveals a *k*_cat_ of 0.41 ± 0.02 substrate · min^−1^ · enzyme^−1^ and a *K_m_* of 16.04 ± 1.28 μM. (D) ^MSMEI_3879^GFP was degraded by 1 μM ClpC1P1P2 with a *k_cat_* of 0.26 ± 0.01 substrate · min^−1^ · enzyme^−1^ and *K_m_* of 2.06 ± 0.43 μM. (E) GFP^MSMEI_3879^ was degraded with a *k_cat_* of 0.09 ± 0.02 substrate · min^−1^ · enzyme^−1^ and a *K_m_* of 18.8 ± 9.6 μM. (F) GFP preceded by the first 13 residues of MSMEI_3879 was degraded with *k*_cat_ of 0.268 ± 0.033 substrate · min^−1^ · enzyme^−1^ and *K_m_* of 19.1 ± 4.9 μM. (G) GFP appended with the last 13 residues of MSMEI_3879 was degraded with a *k*_cat_ of 0.0737 ± 0.0053 substrate · min^−1^ · enzyme^−1^ and *K_m_* of 5.97 ± 1.3 μM. (H) Degradation of 10 μM GFP^ssrA^ by 1 μM ClpC1P1P2 was monitored in the presence and absence of ATP, 10 μM MSMEI_3879, or 10 μM DnaJ1. MSMEI_3879 reduces GFP^ssrA^ degradation to the level observed in the absence of ATP. DnaJ1 does not significantly alter the rate of GFP^ssrA^ proteolysis. Values are averages of three replicates (*n* = 3) ± 1 SD. *P* values were calculated by unpaired two-tailed Student's *t* test. **, *P* < 0.01; n.s., not significant.

We selected several hits from our data set that (i) were expected to be soluble cytosolic proteins, (ii) had M. tuberculosis homologs identified in prior screens for Clp protease interaction partners (Table 3) (21, 23), and (iii) could be readily expressed and purified from E. coli. These included chaperones DnaJ1 and DnaJ2, transcriptional regulators GntR and XRE, magnesium chelatase, and the apparent pseudogene product MSMEI_3879. Five of these (DnaJ1, GntR, XRE, Mg chelatase, MSMEI_3879) produced clear binding curves to ClpC1 by microscale thermophoresis, with *K*_app_ values in the low micromolar range (Fig. S2), which validates the ability of our overall method to identify bona fide interaction partners. We tested whether these hits were recognized as substrates by ClpC1P1P2 *in vitro* and found that only MSMEI_3879 was degraded, with ~75% of the protein hydrolyzed within 40 min (Fig. 7A and B). These *in vitro* results provide preliminary evidence of the substrate status of MSMEI_3879.

**TABLE 3:** ClpC1 interaction partners for which M. tuberculosis homologs were identified in prior ClpC1 interaction studies

UniProt accession no.	Gene name(s)	Protein description	Identified in prior studies	Mean normalized score	Mean no. of PSMs	Data set(s) observed in
A0QQD0	*dnaJ*, *dnaJ1*, MSMEG_0711, MSMEI_0694	Chaperone protein DnaJ1	ClpC1/P2 KD^*a*^	0.676	38.500	WT, EQ, NTD, CORE
A0R203	*atpFH*, *atpF*, *atpH*, MSMEG_4939, MSMEI_4812	ATP synthase subunit b-delta	ClpC1/P2 KD^*a*^	0.568	51.667	WT, EQ, NTD, CORE
I7G417	MSMEI_3879	5-Oxoprolinase	ClpC1/P2 KD^*a*^	0.480	20.500	WT, EQ, NTD, CORE
A0R3L1	MSMEG_5512	Magnesium chelatase	ClpC1/P2 KD,^*a*^ BACTH^*b*^	0.410	28.333	WT, EQ, NTD, CORE
A0QQA8	MSMEG_0688, MSMEI_0671	Aspartate aminotransferase	ClpC1/P2 KD^*a*^	0.292	30.500	WT, NTD, CORE
A0R079	*glnA*, *glnA1*, MSMEG_4290, MSMEI_4189	Glutamine synthetase (GS)	ClpC1/P2 KD^*a*^	0.162	14.000	WT, NTD
A0R617	*pks13*, MSMEG_6392, MSMEI_6224	Polyketide synthase	ClpC1/P2 KD^*a*^	0.160	35.333	WT, NTD
A0R200	*atpD*, MSMEG_4936, MSMEI_4809	ATP synthase subunit beta	ClpC1/P2 KD^*a*^	0.158	11.667	WT, EQ, NTD, CORE
A0R0T1	MSMEG_4497, MSMEI_4386	PhoH family protein	BACTH^*b*^	0.129	9.500	WT, EQ, NTD, CORE
A0QV32	*ffh*, MSMEG_2430, MSMEI_2369	Signal recognition particle protein (fifty-four homolog)	ClpC1/P2 KD^*a*^	0.126	39.500	WT, NTD, CORE
A0R202	*atpA*, MSMEG_4938, MSMEI_4811	ATP synthase subunit alpha	ClpC1/P2 KD^*a*^	0.124	14.667	WT, EQ, NTD
A0QQV4	*gabD2*, MSMEG_0889, MSMEI_0868	Aldehyde dehydrogenase	ClpC1/P2 KD^*a*^	0.123	15.417	WT, EQ, NTD, CORE
A0QQQ1	*sodC*, MSMEG_0835, MSMEI_0816	Superoxide dismutase	ClpC1/P2 KD^*a*^	0.100	6.500	WT, NTD, CORE
A0R0T8	*dnaJ2*, MSMEG_4504, MSMEI_4392	Chaperone protein DnaJ2	ClpC1/P2 KD^*a*^	0.094	21.000	WT, CORE
A0QYD6	*ndh*, MSMEG_3621, MSMEI_3536	NADH dehydrogenase (EC 1.6.99.3)	ClpC1/P2 KD^*a*^	0.093	27.333	WT
A0QR33	*hemL*, MSMEG_0969, MSMEI_0943	Glutamate-1-semialdehyde 2,1-aminomutase (GSA)	ClpC1/P2 KD^*a*^	0.086	18.500	WT
A0QVU2	MSMEG_2695, MSMEI_2629	35-kDa protein	ClpC1/P2 KD^*a*^	0.083	2.500	EQ, NTD
A0QSY1	MSMEG_1642, MSMEI_1603	ABC transporter, ATP-binding protein	ClpC1/P2 KD,^*a*^ BACTH^*b*^	0.083	30.500	WT
A0QX24	*moxR*, MSMEG_3147	ATPase, MoxR family protein	ClpC1/P2 KD^*a*^	0.073	5.500	WT, EQ, NTD
A0QZ40	*tatA*, MSMEG_3887, MSMEI_3797	Sec-independent protein translocase protein TatA	ClpC1/P2 KD^*a*^	0.060	4.500	WT, EQ, NTD
A0R1Z9	*atpC*, MSMEG_4935, MSMEI_4808	ATP synthase epsilon chain	ClpC1/P2 KD^*a*^	0.060	3.000	EQ
A0QT07	*sdhB*, MSMEG_1669, MSMEI_1629	Succinate dehydrogenase, iron-sulfur protein	ClpC1/P2 KD^*a*^	0.059	6.500	EQ
A0QNN8	*pntA*, MSMEG_0110, MSMEI_0106	NAD(P) transhydrogenase, alpha subunit (EC 1.6.1.1)	BACTH^*b*^	0.059	18.000	WT
P71533	*secA1*, MSMEG_1881, MSMEI_1840	Protein translocase subunit SecA 1	ClpC1/P2 KD^*a*^	0.056	28.833	WT, EQ, NTD, CORE
A0QZJ0	MSMEG_4042, MSMEI_3947	Transcriptional regulator, GntR family protein	BACTH^*b*^	0.050	6.000	EQ, CORE
A0R616	MSMEG_6391, MSMEI_6223	Propionyl-CoA carboxylase beta chain	ClpC1/P2 KD^*a*^	0.039	9.500	EQ
Q3L885	*pks*, MSMEG_0408, MSMEI_0398	Polyketide synthase (type I modular polyketide synthase)	ClpC1/P2 KD^*a*^	0.023	43.000	EQ
A0QQY3	MSMEG_0918, MSMEI_0896	Transcriptional regulator, XRE family protein	BACTH^*b*^	0.019	4.667	WT
A0QSL0	MSMEG_1516, MSMEI_1480	Thioredoxin reductase	ClpC1/P2 KD^*a*^	0.012	9.500	WT, NTD, CORE
A0QR26	MSMEG_0962, MSMEI_0936	TetR family protein transcriptional regulator	ClpC1/P2 KD,^*a*^ BACTH^*b*^	0.004	42.000	WT

a Lunge et al. (21).

b Ziemski et al. (23).

The MSMEI_3879 locus encodes a 446-aa product with homology to ATP-hydrolyzing hydantoinase/oxoprolinase enzymes in the hydantoinase A family, which catalyze ring-opening reactions on lactam substrates (70–72). Phylogenetic analysis (Fig. S3) reveals a cluster of orthologs with >90% sequence identity to MSMEI_3879 in several related species, including Mycolicibacterium fortuitum, Mycolicibacterium peregrinum, and Mycolicibacterium brisbanense. More distantly related homologs occur across *Mycobacteriaceae* (73). The closest homolog in M. tuberculosis is OplA, which appears to belong to a separate subclass of enzymes, sharing only 34% sequence identity with MSMEI_3879 and incorporating a C-terminal fusion with a hydantoinase B enzyme.

Notably, *Mycolicibacterium* orthologs of MSMEI_3879 are generally longer, at about 690 aa. Comparison of the M. smegmatis genome to those of other *Mycolicibacterium* species reveals a frameshift caused by a single nucleotide insertion in codon 233 (Fig. S4). The MSMEI_3879 open reading frame arises from an alternative start site at position 242 (with respect to the typical *Mycolicibacterium* start codon), and the resulting polypeptide corresponds to only the latter two-thirds of orthologous hydantoinase/oxoprolinase enzymes. (There is no MSMEG annotation that directly corresponds to MSMEI_3879. The closest analog, MSMEG_3974, describes the entire frameshifted locus.) Comparison of AlphaFold2 (74) predictions of MSMEI_3879 and M. fortuitum AcxA, which share 93% sequence identity and possesses an intact N terminus, suggests that truncation removes a set of surface helices in MSMEI_3879 (Fig. S5). Loss of these helices is predicted to expose hydrophobic residues on two small structural elements that project outward from the body of the protein. In spite of this, MSMEI_3879 expressed well in E. coli, was straightforward to purify and remained stable throughout purification without precipitation or aggregation.

Many Clp protease substrates are recognized by short terminal degron sequences (13, 20, 21, 23). To test whether ClpC1 recognizes MSMEI_3879 by a particular terminus, we engineered green fluorescent protein (GFP) constructs with MSMEI_3879 fused to either the N or C terminus and assayed proteolysis by ClpC1P1P2. Michaelis-Menten analysis of the resulting degradation rates showed that the construct with MSMEI_3879 at the N terminus (^MSMEI_3879^GFP) is degraded with a *k*_cat_ of ≈0.26 substrate · min^−1^ · enzyme^−1^ and a *K_m_* of ≈2 μM (Fig. 7C). For comparison, under the same assay conditions, GFP with an M. smegmatis ssrA tag (GFP^ssrA^) was degraded faster (*k_cat_* ≈ 0.4 substrate · min^−1^ · enzyme^−1^) but with a much higher *K_m_* of ≈16 μM (Fig. 7D), similar to the *K_m_* reported for degradation of GFP^ssrA^ by M. tuberculosis ClpC1 (23). A construct carrying MSMEI_3879 at the C terminus (GFP^MSMEI_3879^) was degraded at a substantially lower rate (*k_cat_* ≈ 0.09 substrate · min^−1^ · enzyme^−1^) and higher *K_m_* (~19 μM) than ^MSMEI_3879^GFP (Fig. 7E). The 9-fold lower *K_m_* of ^MSMEI_3879^GFP suggests that the N terminus of MSMEI_3879 contributes to efficient degradation by ClpC1, but is not the sole determinant of recognition. Neither GFP fusion was degraded by ClpXP1P2 (Fig. S1B), demonstrating that MSMEI_3879 recognition is specific to ClpC1. We also assessed whether sequences at the beginning or end of MSMEI_3879 function as simple degrons by adding its first 13 residues to the beginning of GFP or its final 13 residues to the end of GFP. Neither construct was degraded with as low a *K_m_* or high a *k*_cat_ as ^MSMEI_3879^GFP. While we cannot rule out that longer sequences would be recognized more robustly, our results suggest that efficient recognition involves multivalent contacts on the folded MSMEI_3879 module, likely through the hydrophobic regions exposed by the truncation (Fig. S5). We tested this by assessing proteolysis of “full-length” MSMEI_3879, incorporating a restored N terminus created by removing the nucleotide insertion that causes a frameshift. As expected, this construct was not degraded by ClpC1P1P2 *in vitro* (Fig. S6), confirming that the truncation leads to recognition by ClpC1.

Given the lower *K_m_* for degradation of MSMEI_3879 compared to GFP^ssrA^, we tested whether untagged MSMEI_3879 can compete with GFP^ssrA^ degradation and found that equimolar MSMEI_3879 effectively blocks GFP^ssrA^ proteolysis (Fig. 7F). Inhibition was a specific feature of MSMEI_3879, as DnaJ1, a non-substrate protein from our interactome data set (Fig. 7A), had no effect on degradation (Fig. 7F). Taken together, these data indicate MSMEI_3879 is a novel and robustly degraded ClpC1P1P2 substrate and that its N-terminal sequence contributes to its recognition.

### Chemoinhibition of ClpC1 blocks MSMEI_3879 degradation.

**FIG 8:**
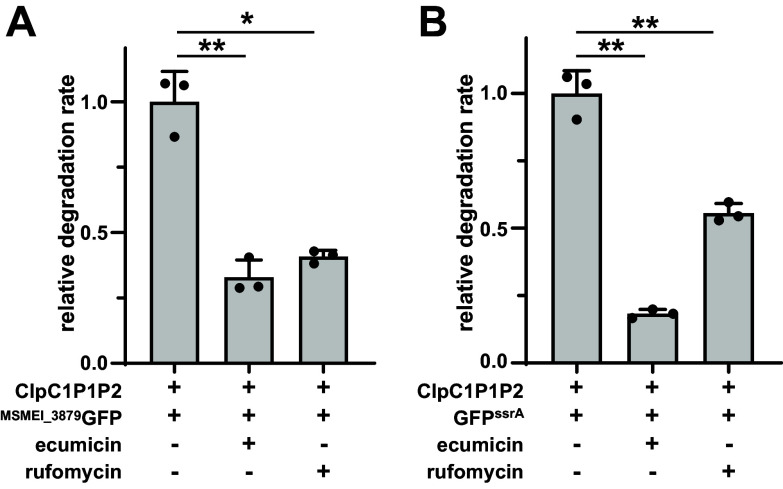
Effect of ClpC1-targeting antibiotics on proteolysis by ClpC1P1P2. Inclusion of 10 μM ECU or RUF inhibits degradation of 10 μM (A) ^MSMEI_3879^GFP and (B) GFP*^ssrA^* by 1 μM ClpC1P1P2 relative to a nontreatment control. Values are averages from three replicates (*n* = 3) ± 1 SD. *P* values were calculated by unpaired two-tailed Student’s *t* test. * and ** represent *P* values of <0.05 and 0.01, respectively.

We sought to assess whether MSMEI_3879 constructs would have utility as reporters for small molecule disruption of ClpC1. We used two well-characterized mycobacterial ClpC1 dysregulators, ecumicin (ECU) and rufomycin (RUF), which bind the ClpC1 NTD and dysregulate the ability of ClpC1 to degrade protein substrates (75–77). We assayed proteolysis of ^MSMEI_3879^GFP and GFP^ssrA^ by ClpC1P1P2 *in vitro* in the presence and absence of 10 μM of each compound. Compared to the untreated control, both ECU and RUF reduced the rate of ^MSMEI_3879^GFP degradation by about 60% (Fig. 8A). Inhibition of GFP^ssrA^ degradation was also observed, but with different magnitude for each compound: ECU inhibited by ~80%, while RUF inhibited by ~45% (Fig. 8B). Taken together, these results demonstrate that known ClpC1 dysregulators inhibit proteolysis of MSMEI_3879 by ClpC1P1P2.

## DISCUSSION

It is now well established that Clp protease components, including ClpC1, are essential for mycobacterial viability (19, 27–31), and these enzymes have consequently emerged as promising antibiotic targets. Here, we sought to better understand the physiological roles of ClpC1P1P2 by identifying partners that interact with full-length and truncated ClpC1 constructs. In total, we found 370 unique cellular proteins that interact with one or more ClpC1 construct. The diversity of the identified ClpC1 interactome is consistent with a multifaceted role for this enzyme in mycobacterial physiology. Moreover, our data align with recent explorations of the M. tuberculosis ClpC1 interactome/degradome (21, 23), as 30 proteins found here possess orthologs identified in those screens.

Several factors likely limit our ability to capture the full breadth of cellular interaction partners. Indeed, some known partners were absent from our data set, including substrates WhiB1 and CarD (30) and the proteolytic adaptor ClpS (23). Our approach was generally biased toward abundant and stably bound partners. Proteolytic substrates—which are actively degraded by the protease and interact with ClpC1 only while ATP is abundant—may be more challenging to detect due to dissociation during capture and wash steps. Clp proteases themselves are known to assemble dynamically and disassemble when substrate or ATP is exhausted (13), which likely occurs during sample preparation. Finally, overexpression presumably creates a stoichiometric excess of ClpC1^FLAG^ over ClpP1P2, which may bias capture toward certain classes of partners.

While ClpC1 likely participates in multiple proteolytic programs in mycobacteria, we note that not all interacting proteins identified here are substrates. While five of six candidates tested directly bind ClpC1 *in vitro* (see Fig. S2 in the supplemental material), only one, MSMEI_3879, proved to be a bona fide substrate (Fig. 7). Non-substrate interaction partners may play a role in regulating ClpC1 activity, may selectively interact with protease-deficient higher-order ClpC1 oligomers (25, 78, 79), or may simply bind nonspecifically under our capture conditions. It is also notable that capture experiments utilizing ClpC1^EQ^, a construct carrying Walker B mutations expected to stabilize interactions with proteolytic substrates (56, 60–62), yielded fewer identified interaction partners (93 proteins) than experiments with ClpC1^WT^ (163 proteins) (Fig. 3, Table 1, and Table 2; see Table S2 in the supplemental material). This suggests that many of these interaction partners are not engaged as the substrates and instead adopt different modes of interaction that are dependent on nucleotide binding, ATP hydrolysis, and perhaps oligomeric state. A diverse set of dynamic interaction partners would plausibly allow mycobacterial cells to nimbly modulate ClpC1 activity across different growth conditions by altering the oligomeric state of ClpC1, changing substrate preferences, or tuning the stability of the entire Clp protease.

ClpC1 is composed of a globular N-terminal domain that binds to substrates and adaptors and an ATPase core consisting of two AAA+ modules that carry out chemomechanical substrate unfolding and translocation (8, 10, 80–82). Surprisingly, our studies identified few interaction partners that bind exclusively to either the NTD or the ATPase core. This suggests that many ClpC1-interacting proteins make multivalent binding to both components. Alternatively, this could result from truncated constructs (ClpC1^NTD^ and ClpC1^CORE^) assembling with endogenous ClpC1 in the cell. Regardless of the mechanism involved, this illustrates that ClpC1 acts through the cooperation of its constituent parts.

Importantly, our interactome analysis led us to identify MSMEI_3879 as a bona fide ClpC1P1P2 substrate (Fig. 7). This protein joins a short list of verified mycobacterial Clp protease substrates (20, 21, 23, 24). MSMEI_3879 is degraded relatively efficiently: a GFP construct carrying an N-terminal MSMEI_3879 fusion was degraded with a *K_m_* ~7-fold lower than that of the model substrate GFP^ssrA^ (13, 17). As expected for this lower *K_m_*, degradation of GFP^ssrA^ was effectively blocked by an equimolar amount of MSMEI_3879 (Fig. 7F). Our experiments indicate that recognition of MSMEI_3879 by ClpC1 likely involves elements exposed as a result of its N-terminal truncation, due to a unique frameshift mutation (Fig. S4 and S5). Indeed, ClpC1 has been proposed to selectively recognize some substrates via hydrophobic regions and disordered termini (21). It is unclear whether MSMEI_3879 carries out any significant enzymatic function, or whether its proteolysis by ClpC1 plays any role in M. smegmatis physiology. Regardless, MSMEI_3879-based substrates may serve as useful tools for probing ClpC1P1P2 activity and dysregulation. As a proof of concept, we show that known ClpC1-targeting antimicrobials inhibit ^MSMEI_3879^GFP proteolysis *in vitro* (Fig. 8). Similar substrates could serve as the basis for cell-based screens to identify novel ClpC1-targeting antibiotics.

## MATERIALS AND METHODS

### Plasmid and strain construction.

Full-length ClpC1 (ClpC1^WT^), the ClpC1 NTD alone (aa 1 to 147 [ClpC1^NTD^]), and ClpC1 lacking the NTD (aa 158 to 848 [ClpC1^CORE^]) were amplified from Mycolicibacterium smegmatis (strain ATCC 700084/MC^2^155) genomic DNA (ATCC) and cloned into a modified episomal pNIT expression vector (83), followed by a C-terminal 3×FLAG tag. Mutations to Walker B motifs in the D1 (E288Q) and D2 (E626Q) rings of ClpC1^WT^ were introduced by sequential polymerase chain reactions, yielding an ATPase inactive “trap” variant (ClpC1^EQ^) (56). Plasmid sequences were verified by Sanger sequencing (Genewiz).

### *In vivo* substrate trapping.

Plasmids encoding M. smegmatis ClpC1 constructs were electroporated into M. smegmatis (ATCC 700084/MC^2^155) at 5 kV in a MicroPulser (Bio-Rad). Liquid starter cultures were made in Middlebrook broth base (HiMedia) supplemented with 0.2% (vol/vol) glycerol (Fisher Scientific), 0.2% (wt/vol) glucose (TCI), and 0.05% (vol/vol) Tween 80 and then (with orbital shaking) grown for 60 h at 37°C. At a starting *A*_600_ of 0.05, starter cultures were subcultured into 200 mL of fresh medium. The cultures were then grown at 37°C until the mid-log phase (*A*_600_ ≈ 0.6 to 1.0), at which point expression was induced by 28 mM ε-caprolactam (Sigma-Aldrich) for 20 h. All constructs continued to grow similarly over the course of expression. For cell harvesting, centrifugation was carried out at 9,000 × *g* for 20 min at 4°C and pellets were resuspended in 5 mL of lysis buffer (25 mM HEPES, 10 mM magnesium chloride, 200 mM potassium chloride, and 0.1 mM EDTA, supplemented with 10 mM ATP [pH 7.5]). Cell lysis was done in a microfluidizer (Microfluidics), and subsequent clarification of lysates was performed at 16,000 × *g* for 30 min at 4°C. Cell supernatants were stored at −80°C. A Bradford assay (Bio-Rad) was performed to estimate total protein content.

### Coimmunoprecipitation.

Forty microliters of EZView Red anti-FLAG M2-affinity gel beads (Sigma-Aldrich) was equilibrated and washed twice in 0.5 mL lysis buffer by centrifugation at 8,200 × *g* for 30 s. To pull down the expressed 3×FLAG-tagged M. smegmatis ClpC1 constructs and interacting proteins, the equilibrated beads were incubated with 1 mL of lysate for 1 h at 4°C with gentle agitation. After incubation, the bead-lysate slurry was spun at 8,200 × *g* for 30 s and the bead pellet was subsequently washed with lysis buffer (containing 10 mM ATP). To elute, the washed beads were then mixed with 20 μL of 2× Laemmli sample buffer (10% glycerol, 4% SDS, 167 mM Tris-HCl, 0.02% bromophenol blue) in the absence of reducing agent and boiled for 5 min. Samples were then vortexed briefly and centrifuged, and the supernatant containing the eluate was stored at −80°C prior to further analysis. As a negative control, lysates of cells containing empty pNIT vector were processed by the same workflow.

### Mass spectrometry sample preparation.

Proteins in the eluate were analyzed on a 6 to 15% SDS-PAGE gradient gel. Each lane was diced, and gel pieces were put into 1.5-mL microcentrifuge tubes. The samples were prepared by adapting standard protocols for in-gel mass spectrometry sample preparation (84). Incubation with 10 mM dithiothreitol (DTT) was carried out for 45 min at 55°C in order to reduce disulfide bonds. Afterwards, carbamidomethylation of cysteines was with 55 μM iodoacetamide for 30 min at room temperature (in the dark). Gel pieces were washed with gel wash buffer (25 mM ammonium bicarbonate and 50% acetonitrile) and dehydrated with 100% acetonitrile prior to drying in a SpeedVac vacuum centrifuge (Thermo). Trypsin digestion was carried out in 25 mM ammonium bicarbonate containing 10 μg mL^−1^ MS-grade trypsin protease (Pierce), and the mixture was incubated overnight at 37°C. Peptides were subsequently extracted by a two-step process, which was repeated twice: incubation of gel pieces with 5% formic acid (Sigma-Aldrich), followed by 100% acetonitrile. To remove residual salts, the peptide samples were desalted using a HyperSep C_18_ column (Thermo Fisher Scientific) according to a previously described procedure (84, 85). C_18_ column-eluted samples were dried by vacuum centrifugation and frozen prior to mass spectrometry.

### LC-MS/MS.

Liquid chromatography tandem mass spectrometry (LC-MS/MS) analysis was performed using a Q Exactive Orbitrap interfaced with Ultimate 3000 Nano-LC system (Thermo Fisher Scientific). Trypsin-digested samples were loaded on an Acclaim PepMap rapid-separation liquid chromatography (RSLC) column (75-μm by 15-cm nanoViper) using an autosampler. Analysis of samples was done using a 150-min gradient running from 2% to 95% buffer B (0.1% formic acid in acetonitrile) in buffer A (0.1% formic acid in water) at a flow rate of 0.3 μL min^−1^. MS data acquisition was done using a data-dependent top10 method, with the most abundant precursor ions from the survey scan chosen for higher-energy collisional dissociation (HCD) fragmentation using stepped normalized collision energies of 28, 30, and 35 eV. Survey scans were acquired at a resolution of 70,000 at *m*/*z* 200 on the Q Exactive. LS-MS/MS data were collected in independent biological triplicates.

### MS data analysis.

For proteomic analysis, extraction of raw data was performed in the Proteome Discoverer software suite (version 1.4; Thermo Fisher Scientific). The raw data were searched against Mycolicibacterium smegmatis (strain ATCC 700084/MC^2^155) UniProt Reference Proteome (Proteome ID UP000000757) using Sequest HT (University of Washington and Thermo Fisher Scientific). Iodoacetamide-mediated cysteine carbamidomethylation was set as a static modification. Precursor mass tolerance was set at 10 ppm, while allowing for fragment ion mass deviation of 0.6 Da for the HCD data, and full trypsinization with a maximum of two missed cleavages. Peptide-spectrum match (PSM) validation was done using Percolator, with false-discovery rates (FDRs) of 1% and 5% for stringent and relaxed validation, respectively. Gene Ontology (GO) annotation analysis on the data sets was performed using the Blast2GO software suite (64). Some GO annotation terms were binned into a finite number of final terms.

### Expression and purification of recombinant proteins.

N-terminally H_7_-SUMO-tagged ClpC1 (with or without a C-terminal FLAG tag), N-terminally H_7_-SUMO-tagged ClpX, and C-terminal H_6_-tagged ClpP1 and ClpP2 were cloning into pET22b-derived vectors using Gibson Assembly (86). N-terminal H_7_-SUMO-tagged putative substrates and interaction partners were obtained as synthetic gene constructs (Twist Bioscience) and cloned into pET29b(+). All constructs were expressed in E. coli ER2566 (NEB). Cultures were grown in 1.5×YT at 37°C to exponential phase (*A*_600_ of 0.8 to 1.0), and overexpression was induced with 0.5 mM isopropyl-β-d-thiogalactopyranoside (IPTG), followed by incubation at 30°C for 4 h. Cells were harvested at 4,000 × *g* for 30 min, and pellets were resuspended in 25 mL His tag lysis buffer (25 mM HEPES [pH 7.5], 300 mM NaCl, 10 mM imidazole [pH 7.5], 10% glycerol), supplemented with 1 mM phenylmethylsulfonyl fluoride (PMSF) and 100 μL of EDTA-free protease inhibitor cocktail (Thermo Fisher). After sonication and clarification at 15,000 × *g* for 30 min, lysates were loaded onto a Ni-nitrilotriacetic acid (NTA) column (MCLAB), washed with 25 mM imidazole, and eluted in 300 mM imidazole. The eluate was spin concentrated (10,000-molecular-weight-cutoff [MWCO]; Amicon, MilliporeSigma) at 4,000 × *g*. Protein samples were further purified by anion-exchange chromatography (Source 15Q 10/100; Cytiva). H_7_-SUMO tags were removed by incubation with Saccharomyces cerevisiae SUMO protease Ulp1 (87) or left intact for microscale thermophoresis studies. Constructs were additionally purified by gel filtration (HiLoad 16/600 Superdex 200; Cytiva) into protein degradation buffer (25 mM HEPES, 200 mM potassium chloride, 10 mM magnesium chloride, 0.1 mM EDTA [pH 7.5]).

### *In vitro* assays.

*In vitro* degradation assays containing 1 μM ClpC1 (hexamer), 1 μM ClpP1 (tetradecamer), 1 μM ClpP2 (tetradecamer), and 10 μM substrate were performed in ClpC1 protein degradation buffer. All degradation assays were carried out in the presence of 50 μM activator peptide *Z*-Leu-Leu-Nva-CHO (benzyloxycarbonyl-l-leucyl-l-leucyl-l-norvalinal) (12, 13), with a total of 15 mM ATP, along with an ATP regeneration system consisting of 187.5 U mL^−1^ pyruvate kinase and 50 mM phosphoenolpyruvate (Sigma). For gel degradation assays, 14-μL aliquots were taken at each time point, mixed with 7 μL 2× Laemmli sample buffer (containing 10% β-mercaptoethanol), and analyzed by SDS-PAGE. Gels were stained by 0.1% Coomassie brilliant blue and quantified by ImageJ (88). Plate reader assays were carried out on a Tecan Spark instrument. Degradation of GFP-substrate fusions was monitored by loss of 511-nm emission following excitation at 450 nm. ATPase assays utilized 1 μM ClpC1, 10 mM ATP, and an NADH-coupled ATP regeneration system (89). Consumption of ATP was followed by monitoring the decrease in NADH absorbance at 340 nm. Microscale thermophoresis was performed in a Monolith NT.115 (NanoTemper) using 0.1 μM ^H7-SUMO^ClpC1^FLAG^ (hexamer) and 0.1 μM His Lite OG488-Tris-NTA-Ni dye (AAT Bioquest). Data were fit to a Hill-form binding equation in Prism (GraphPad).

### CRISPRi assays.

Integrative plasmid PLJR962 (Addgene) (90), which encodes dCas9 machinery driven by an anhydrotetracycline (aTc)-responsive promoter for CRISPR interference (CRISPRi) in M. smegmatis, was modified by Gibson Assembly to contain either a nontargeting (GAGACGATTAATGCGTCTCG) or *clpC1*-targeting (ATGAGCGCGTCGTCGTCGCCGAA) small guide RNA (sgRNA). Versions of these plasmids were constructed by Gibson Assembly to contain a secondary *clpC1* (or *clpC1^FLAG^*, *clpC1^EQ^*^,^*^FLAG^*) locus downstream of the CRISPRi machinery. The plasmid-borne copy of *clpC1* was modified to incorporate strategic codon substitutions to the sgRNA-targeted region that prevent sgRNA binding and thus escape transcriptional knockdown. Plasmids were transformed into M. smegmatis, and growth was monitored on Middlebrook agar plates at 37°C at various levels of aTc induction.

### Data availability.

The mass spectrometry data from this work have been submitted to the ProteomeXchange Consortium via the PRIDE partner repository (91) and assigned the identifier PXD030385.
