# Supplementary material for: Interactome Analysis Identifies MSMEI_3879 as a Substrate of Mycolicibacterium smegmatis ClpC1
Source: Microbiol Spectr. 2023 Jun 21;11(4):e04548-22. doi: 10.1128/spectrum.04548-22 (PMC10433963; doi:10.1128/spectrum.04548-22)
Supplement: Supplemental file 3 — Supplemental material. Download spectrum.04548-22-s0003.pdf, PDF file, 14.9 MB [file spectrum.04548-22-s0003.pdf]

# SUPPLEMENTAL MATERIAL

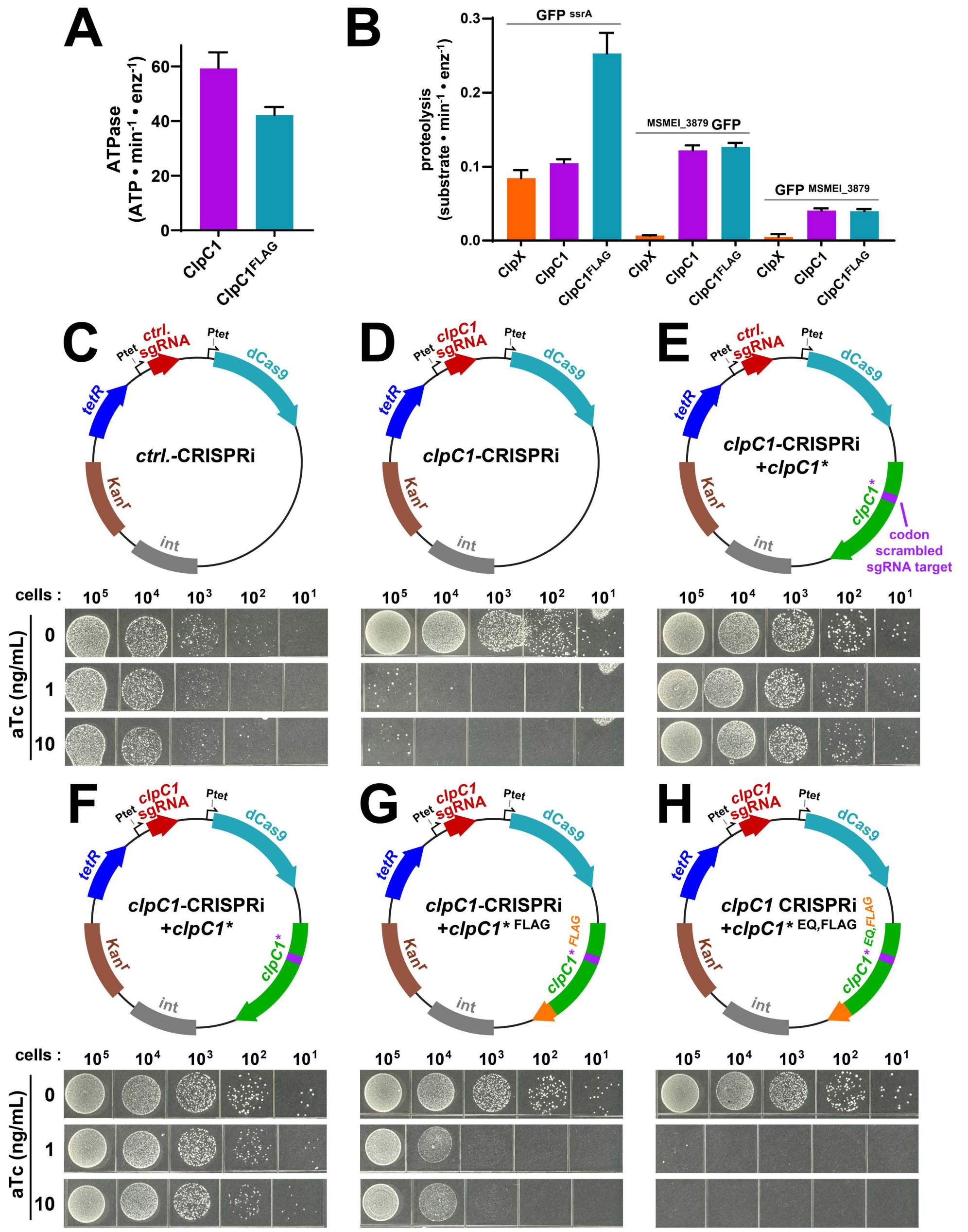


**Supplemental Figure S1: ClpC1-FLAG is functional. (A)** C-terminally FLAG-tagged ClpC1 has a ~30% lower ATPase rate than untagged ClpC1. **(B)** Degradation of 10 µM $GFP^{ssrA}$ , $^{MSMEI\_3879}GFP$, or $GFP^{MSMEI\_3879}$ by 1 µM ClpXP1P2, ClpC1P1P2, or $ClpC1^{FLAG}P1P2$ demonstrates that ClpX does not recognize MSMEI_3879 constructs, while $ClpC1^{FLAG}$ retains the ability to recognize these substrates. (**C – H**) *M. smegmatis* was transformed with an integrative CRISPRi plasmid carrying either (**C, E**) a non-targeting control sgRNA or (**D, F, G, H**) a ClpC1-targeting sgRNA. Additionally, some plasmids carried a supplemental copy of (**E, F**) wild-type *clpC1*, (**G**) *clpC1* with a C-terminal FLAG-tag, or (**H**) *clpC1* with both a FLAG-tag and inactivating mutations to ATPase active sites ("EQ"). All supplemental *clpC1* loci incorporate codon substitutions that escape sgRNA targeting, indicated by asterisks.

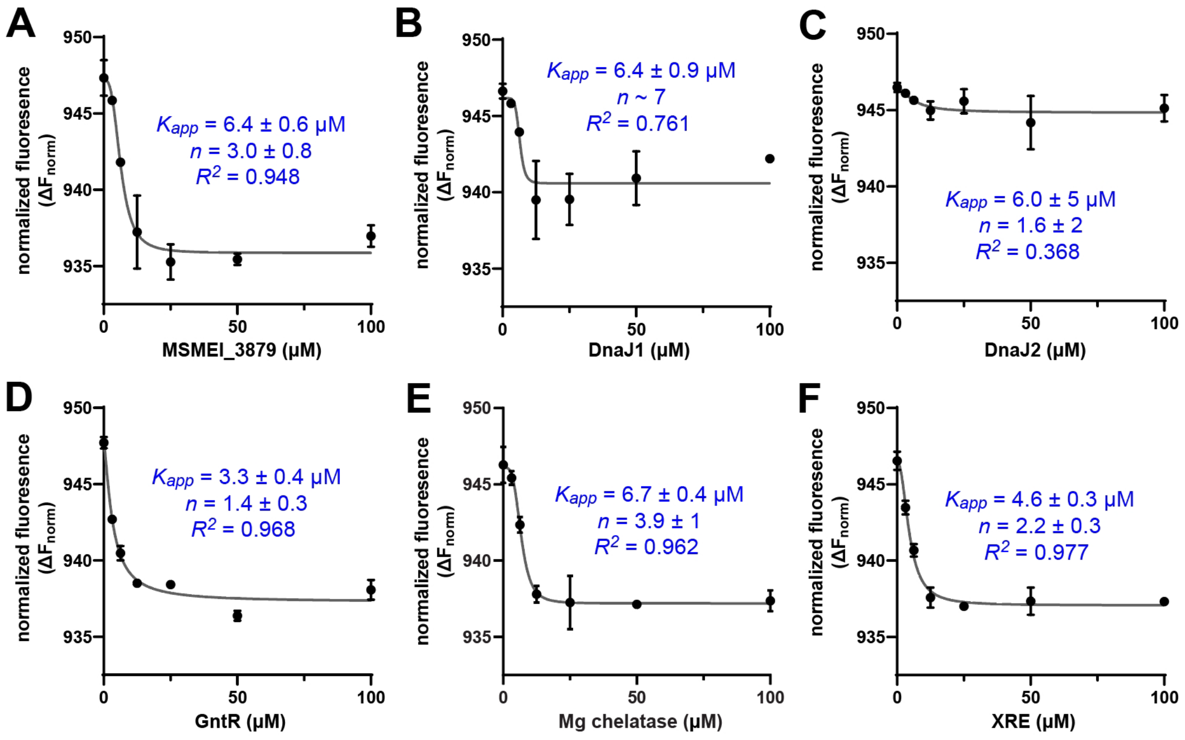


**Supplemental Figure S2: Candidate interaction partners bind to ClpC1 *in vitro*.** Binding of 0.1 µM $^{7xHis\text{-}SUMO}$ClpC1$^{FLAG}$ to purified (**A**) MSMEI_3879, (**B**) DnaJ1, (**C**) DnaJ2, (**D**) GntR, (**E**) Mg chelatase, or (**F**) XRE was assayed by microscale thermophoresis in the presence of 1 mM ATPγS. Data were fit to a Hill binding equation, and fit parameters are shown.

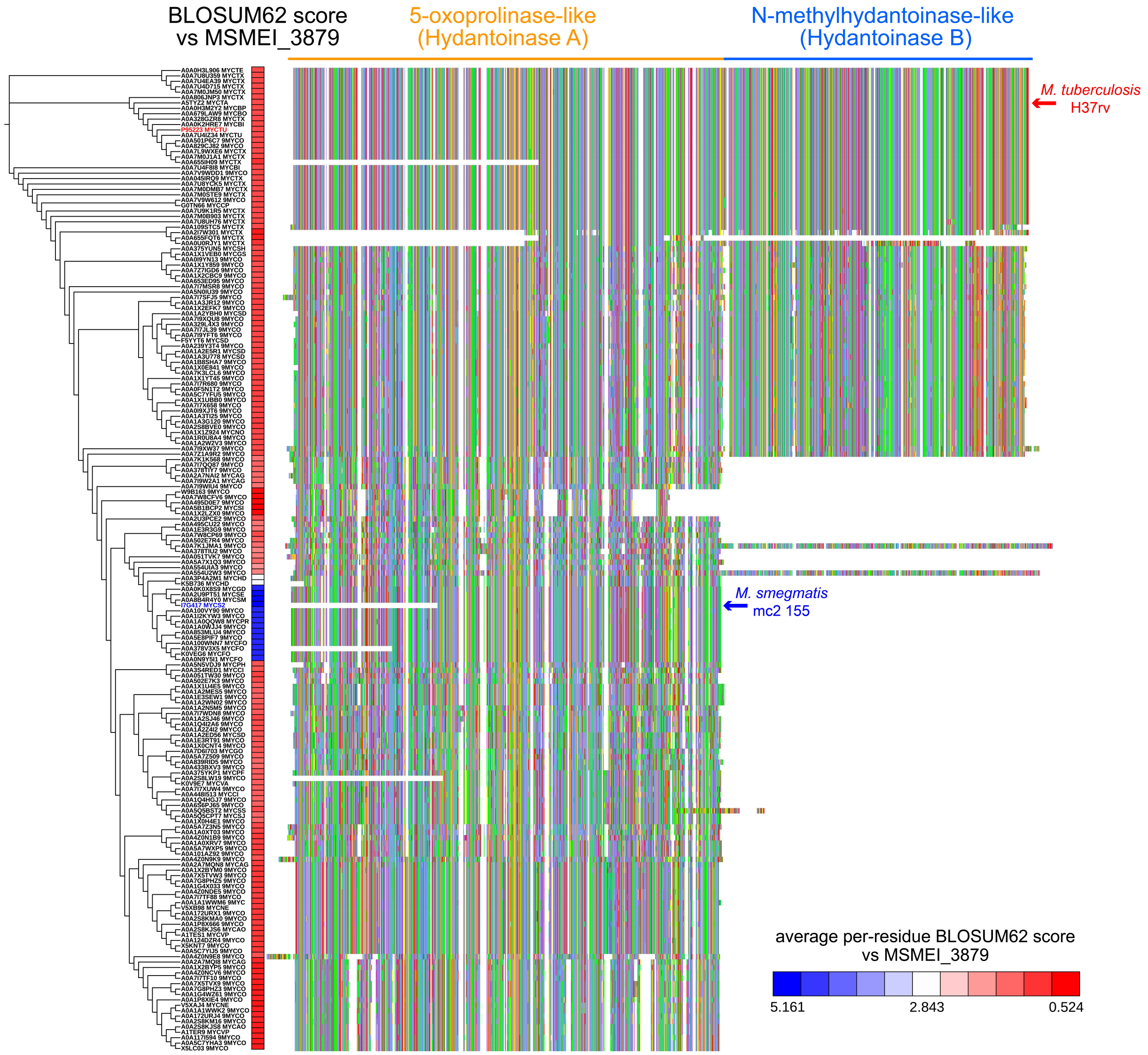


**Supplemental Figure S3: Phylogenetic analysis of MSMEI_3879 homologs.** Homologs of MSMEI_3879 in Mycobacteriaceae were compiled, aligned, and used to generate a phylogentic tree. The average per-residue BLOSUM62 score of each sequence to MSMEI_3879 is shown as a heatmap strip. A representation of the sequence alignment, colored according to residue type, is shown alongside the tree. *M. smegmatis* MSMEI_3879 (blue) and *M. tuberculosis* OplA (red) are indicated.

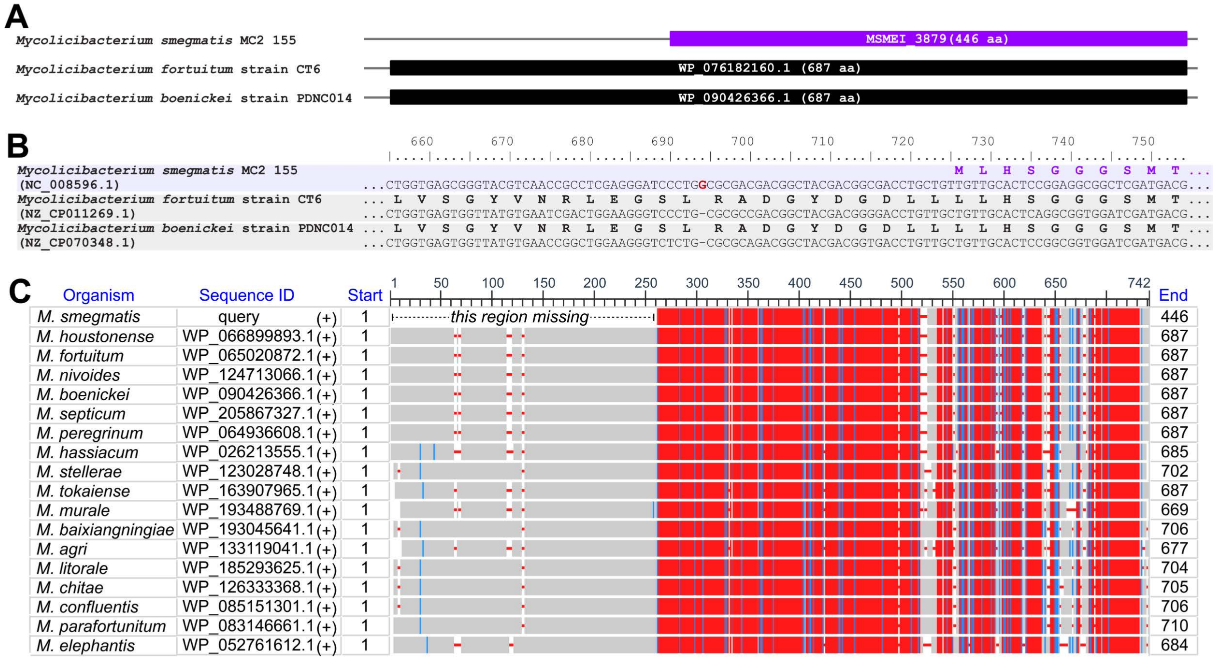


**Supplemental Figure S4: Comparison of MSMEI_3879 to full-length homologs. (A)** Genetic context of *M. smegmatis* MSMEI_3879 compared to the equivalent genomic region in the indicated *Mycolicibacterium* species. **(B)** Sections of genomic sequence show the beginning of the disrupted *M. smegmatis* hydantoinase/oxoprolinase gene and the analogous regions in the intact genes of *M. fortuitum* and *M. boenickei*. Numbering is given from the beginning of the normal intact open reading frame. Amino acid translations of MSMEI_3879 (purple) and hydantoinase/oxoprolinase orthologs (black) are shown. A single nucleotide insertion in the *M. smegmatis* genome (red) disrupts the gene. **(C)** NCBI protein BLAST was used to align MSMEI_3879 (top) with full-length hydantoinase/oxoprolinase orthologs from select *Mycolicibacterium* species. The upstream region missing in MSMEI_3879 is indicated.

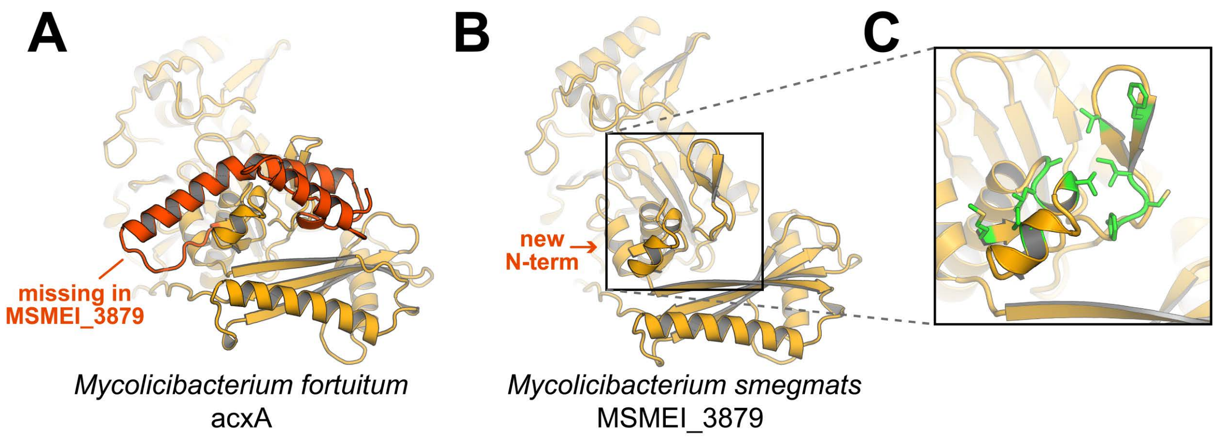


**Supplemental Figure S5: Structure prediction of MSMEI_3879.** AlphaFold2 structure predictions of **(A)** *M. fortuitum* axcA, which lacks an N-terminal truncation, and **(B)** *M. smegmatis* MSMEI_3879 are shown as cartoons. The region missing due to truncation of MSMEI_3879 is shown as orange in acxA. **(C)** Loss of this region exposes hydrophobic residues (green) on structured elements that project from the folded core of the protein.

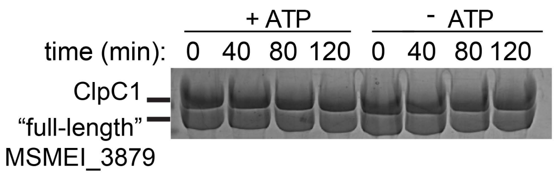


**Supplemental Figure S6: "Full-length" MSMEI_3879 is not degraded by ClpC1P1P2.** Proteolysis of purified "full-length" MSMEI_3879, with restored N-terminus, was assayed by SDS-PAGE as in Figure 7B.
